# Comparison of Untargeted Metabolomic Profiling vs Traditional Metabolic Screening to Identify Inborn Errors of Metabolism

**DOI:** 10.1001/jamanetworkopen.2021.14155

**Published:** 2021-07-12

**Authors:** Ning Liu, Jing Xiao, Charul Gijavanekar, Kirk L. Pappan, Kevin E. Glinton, Brian J. Shayota, Adam D. Kennedy, Qin Sun, V. Reid Sutton, Sarah H. Elsea

**Affiliations:** 1 Department of Molecular and Human Genetics, Baylor College of Medicine, Houston, Texas; 2 Baylor Genetics, Houston, Texas; 3 Metabolon, Inc, Durham, North Carolina; 4 Now with Owlstone Medical, Inc, Research Triangle Park, North Carolina; 5 Now with Division of Medical Genetics, Department of Pediatrics, University of Utah, Salt Lake City

## Abstract

**Importance:**

Recent advances in newborn screening (NBS) have improved the diagnosis of inborn errors of metabolism (IEMs); however, many potentially treatable IEMs are not included on NBS panels, nor are they covered in standard, first-line biochemical testing.

**Objective:**

To examine the utility of untargeted metabolomics as a primary screening tool for IEMs by comparing the diagnostic rate of clinical metabolomics with the recommended traditional metabolic screening approach.

**Design, Setting, and Participants:**

This cross-sectional study compares data from 4464 clinical samples received from 1483 unrelated families referred for trio testing of plasma amino acids, plasma acylcarnitine profiling, and urine organic acids (June 2014 to October 2018) and 2000 consecutive plasma samples from 1807 unrelated families (July 2014 to February 2019) received for clinical metabolomic screening at a College of American Pathologists and Clinical Laboratory Improvement Amendments–certified biochemical genetics laboratory. Data analysis was performed from September 2019 to August 2020.

**Exposures:**

Metabolic and molecular tests performed at a genetic testing reference laboratory in the US and available clinical information for each patient were assessed to determine diagnostic rate.

**Main Outcomes and Measures:**

The diagnostic rate of traditional metabolic screening compared with clinical metabolomic profiling was assessed in the context of expanded NBS.

**Results:**

Of 1483 cases screened by the traditional approach, 912 patients (61.5%) were male and 1465 (98.8%) were pediatric (mean [SD] age, 4.1 [6.0] years; range, 0-65 years). A total of 19 families were identified with IEMs, resulting in a 1.3% diagnostic rate. A total of 14 IEMs were detected, including 3 conditions not included in the Recommended Uniform Screening Panel for NBS. Of the 1807 unrelated families undergoing plasma metabolomic profiling, 1059 patients (58.6%) were male, and 1665 (92.1%) were pediatric (mean [SD] age, 8.1 [10.4] years; range, 0-80 years). Screening identified 128 unique cases with IEMs, giving an overall diagnostic rate of 7.1%. In total, 70 different metabolic conditions were identified, including 49 conditions not presently included on the Recommended Uniform Screening Panel for NBS.

**Conclusions and Relevance:**

These findings suggest that untargeted metabolomics provided a 6-fold higher diagnostic yield compared with the conventional screening approach and identified a broader spectrum of IEMs. Notably, with the expansion of NBS programs, traditional metabolic testing approaches identify few disorders beyond those covered on the NBS. These data support the capability of clinical untargeted metabolomics in screening for IEMs and suggest that broader screening approaches should be considered in the initial evaluation for metabolic disorders.

## Key Points

**Question:**

Is untargeted metabolomic profiling associated with a significant increase in the diagnostic rate of screening for inborn errors of metabolism (IEMs) compared with the traditional metabolic screening approach?

**Findings:**

This cross-sectional analysis of 4464 traditional metabolic screening samples and 2000 plasma metabolomic screening samples received at a clinical biochemical laboratory between July 2014 and February 2019 found a 1.3% diagnostic rate for traditional metabolic screening, whereas clinical metabolomics supported diagnosis in 7.1% of cases, providing an approximately 6-fold higher diagnostic rate in screening for IEMs and identifying more disorders and more disease types compared with the traditional screening approach.

**Meaning:**

With expanded newborn screening available in all states in the US, a broader approach to primary screening for IEMs is needed, and these data support the application of untargeted clinical metabolomics to serve as a primary screening approach.

## Teaser

This cross-sectional study examines the utility of untargeted metabolomics as a primary screening tool for inborn errors of metabolism by comparing the diagnostic rate of clinical metabolomics with the recommended traditional metabolic screening approach.

## Introduction

Early detection and diagnosis of inborn errors of metabolism (IEMs) are imperative because many conditions are amenable to treatment, and sequelae that develop before therapy is initiated are often irreversible.^1,2,3^ Newborn screening (NBS) is the first-tier assessment of IEMs and aims to identify apparently healthy newborns with serious conditions to improve neonatal and lifelong health outcomes. As a result of advances in tandem mass spectrometry technology, NBS has expanded, and 49 metabolic conditions, including 25 core conditions and 24 secondary conditions, are currently listed on the Recommended Uniform Screening Panel (RUSP) with variations in different states. However, many treatable IEMs are not included on the RUSP; thus, individuals with signs and symptoms of an IEM still require further testing, even when the NBS findings are normal.

The standard diagnostic approach for IEMs involves the recognition of symptoms and routine laboratory abnormalities followed by biochemical and/or genetic testing. Clinically, IEMs have a wide range of presentations, from acute to chronic, involving virtually any tissue or organ of the human body, and clinical manifestations may arise at different periods, ranging from neonatal to late adulthood.^4,5^ Therefore, the nonspecific nature of these sequelae often presents diagnostic challenges. Laboratory assessments of metabolic disorders may be prolonged because the standard approach involves multiple, and often sequential, targeted biochemical tests.^1,6^ Optimal evaluation of patients presenting with nonspecific neurological findings, including intellectual disability, global developmental delays, or autism spectrum disorder, follows recommendations provided by the American Academy of Neurology, the American Academy of Pediatrics, and the National Academy of Clinical Biochemistry, stating that first-line screening for IEMs should be considered in the initial evaluation.^7,8,9^ The traditional metabolic screening approach includes a trio of biochemical analyses: plasma amino acids (PAA), plasma acylcarnitine profile (ACP), and urine organic acids (UOA). NBS targets 43 of 49 disorders diagnosable by this trio of tests, and, to our knowledge, no recent studies have assessed the current diagnostic rate for this trio of tests in the era of expanded NBS (eTable 1 in the Supplement).

Clinical metabolomics, a broad, comprehensive analysis of small molecules in body fluids, uses liquid chromatography–coupled mass spectrometry and is sensitive, with diverse coverage.^10,11^ This method is capable of detecting multiple metabolites with varying chemical properties in a single test for the global analysis of perturbations in biochemical pathways that would otherwise require multiple targeted tests. The potential application of untargeted metabolomics to clinical diagnostic screening for IEMs in the precision medicine era is supported by multiple published studies.^12,13,14,15^ To test our hypothesis that untargeted metabolomics could function as a primary initial screen for IEMs, we assessed data from a large-scale clinical analysis of 2000 clinical metabolomic samples to determine the diagnostic rate compared with that of traditional metabolic screening (PAA, ACP, and UOA).

## Methods

This study was approved by the Baylor College of Medicine institutional review board with a waiver of informed consent because this study was performed to assess effectiveness of clinical laboratory testing, involved no more than minimal risk to the participants, could not be performed practicably without the waiver, and would not adversely affect the rights and welfare of the participants. All laboratory testing reported in this study was performed at the request of the referring clinician and in the process of clinical care of the patient. This study follows the Strengthening the Reporting of Observational Studies in Epidemiology (STROBE) reporting guideline.

### Patient Population

This cross-sectional study compared data from 2 different cohorts: a traditional metabolic screening cohort and a metabolomic screening cohort defined by the tests ordered in our clinical laboratory. The traditional screening cohort included samples from 1488 consecutive patients from 1483 unrelated families (4464 clinical samples collected in total, 1 sample each for 3 tests) referred for clinical biochemical testing from June 2014 through October 2018. All individuals underwent PAA, ACP, and UOA analyses from samples collected on the same day. Most samples received were not submitted with a clinical indication or phenotyping information; however, it is likely that most were obtained to screen for IEMs or follow-up of equivocal results from other testing, including NBS. The clinical metabolomic screening cohort consisted of plasma samples from 1807 unrelated families (2000 clinical samples collected in total) assessed by untargeted metabolomic profiling between July 2014 and February 2019, including 158 families that were also analyzed by traditional screening approach. Only the initial plasma metabolomic samples collected from the probands were included in our analysis pipeline, as the metabolites of the repeated specimens may not accurately reflect the real disease states because of dietary or medication management. Most of the clinical metabolomic samples were submitted with detailed descriptions of the patient’s clinical phenotype.

A request for traditional targeted biochemical testing and/or metabolomic testing was made solely at the discretion of the referring health care practitioner with no required or potential criteria and no filtering by the laboratory reflecting the unbiased comparison of these approaches. All testing was performed in the clinical biochemical genetics laboratory at Baylor Genetics. Untargeted clinical metabolomics was performed in collaboration with Metabolon, Inc. Both laboratories are certified by the College of American Pathologists and in compliance with the Clinical Laboratory Improvement Amendments.

### Procedures

Targeted clinical biochemical tests (PAA, ACP, and UOA) were performed as described previously (eAppendix 1 in the Supplement).^13^ Clinical metabolomic profiling of plasma was developed and performed through a collaboration between Baylor College of Medicine, Baylor Genetics, and Metabolon, Inc, and was performed as previously described (eAppendix 2 in the Supplement).^10,11,13,16,17,18,19,20^

### Analysis Pipeline and Diagnostic Criteria

**Figure 1.**
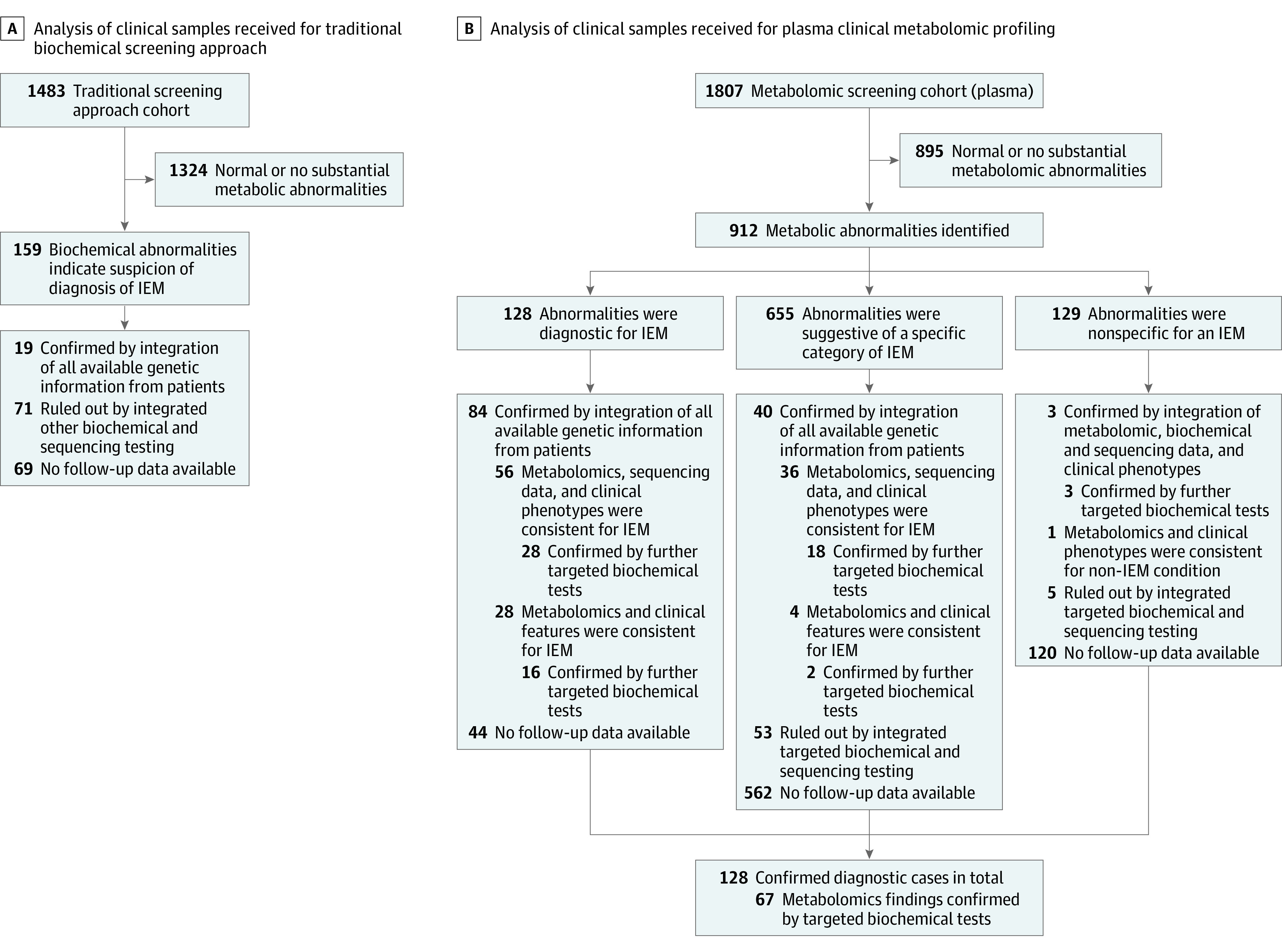
Assessment of Clinical Samples Referred for Metabolic Screening A, Analysis of clinical samples received for traditional biochemical screening. Patients referred for targeted traditional screening approaches including plasma amino acids, urine organic acids, and plasma acylcarnitine profile between June 2014 and October 2018 were reviewed and all associated patient data evaluated. Numbers represent unrelated families. B, Analysis of clinical samples received for plasma untargeted clinical metabolomics. Patients referred for plasma metabolomic profiling between July 2014 and February 2019 were evaluated to provide a comprehensive and in-depth analysis. Numbers represent unique, unrelated families. IEMs indicates inborn errors of metabolism.

Testing results from PAA, ACP, and UOA analyses were reviewed, analyzed, and interpreted by board-certified clinical biochemical geneticists through a standard data analysis pipeline (Figure 1A). A case was classified as abnormal when substantial abnormal results were detected in a single sample, such as the presence, reduction, or elevation of specific diagnostic markers for known metabolic disorders, or when combined results of PAA, ACP, and/or UOA analyses suggested a pattern indicating a possible IEM. When no substantial abnormalities were detected, the patient was not further considered in the analysis pipeline. An abnormal case was classified as diagnosed after in-depth analysis considering all other data collected from the patient. To facilitate this analysis, all available clinical and genetic details were considered, including clinical phenotype, metabolomic profiling analysis, targeted biochemical diagnostic testing results, and molecular testing results, which may include exome sequencing, chromosome microarray, cytogenetic analysis, fluorescent in situ hybridization, targeted gene sequencing panels, mitochondrial DNA sequencing, and/or mitochondrial DNA copy number analysis. Data were integrated, reviewed, and interpreted by an interdisciplinary analysis team according to current American College of Medical Genetics and Genomics and Association for Molecular Pathology guidelines. A case was considered confirmed only when available clinical presentation, genetic testing results, and follow-up evaluations were consistent with the biochemical findings for a diagnosis of IEMs. Cases with a definitive diagnosis were pooled, and the positive diagnostic rate was determined.

To assess clinical metabolomic screening outcomes, a similar global meta-analysis pipeline was applied to provide an in-depth analysis for every clinical sample. The cases in the metabolomic cohort with substantial biochemical abnormalities were divided into different categories according to whether the biochemical profiles were (1) specific for, (2) indicative of, or (3) nonspecific for an IEM (Figure 1B). Further in-depth analysis of these categories by integrating other findings were applied to confirm or exclude the diagnosis for each case (the same pipeline as described already for the traditional screening approach). Of note, some cases were not classified as positive in this analysis even though metabolomic profiling data were consistent with a disease pattern; this underestimate of final positive diagnostic rate was due either to metabolomic data not being definitively diagnostic for the specific condition and/or the absence of other testing to confirm the diagnosis. As a reference laboratory, follow-up and/or comprehensive clinical data were not available for all patients tested.

### Statistical Analysis

Data were collected from samples received between July 2014 and February 2019. Descriptive statistics were used to characterize the clinical data using Excel for Mac spreadsheet software version 16.30 (Microsoft). Available current clinical data from all referred cases were gathered and analyzed from September 2019 to August 2020.

## Results

### Traditional Metabolic Screening for IEMs

#### Demographic Characteristics of Clinical Cases

Traditional metabolic screening performed on samples from 1483 families submitted to the biochemical genetics laboratory for traditional metabolic screening was primarily pediatric (1465 children [98.8%]; mean [SD] age, 4.1 [6.0] years; range, 0-65 years), including 912 (61.5%) male patients. The cohort included 1016 children younger than 5 years (68.5%), 449 children and adolescents aged 5 to 21 years (30.3%), and 18 individuals older than 21 years (1.2%) (Table 1). Limited clinical information was provided for each patient; however, most samples were submitted because of nonspecific neurological presentations, whereas others were submitted for the follow-up of abnormal NBS results.

**Table 1.** Cohort Demographic Characteristics and Comparison of Diagnostic Rate Between Clinical Metabolomics and Traditional Metabolic Screening Approaches

Screening methods	Screening cohort, No. of samples	Patients, No. (%)			Overall screening results^a^		
		Male	Age <21 y	Neurological	Positive cases, No.	Identified IEMs, No.	Positive diagnostic rate, %
Traditional approach (plasma, urine) (n = 1483 families)	4464	912 (61.5)	1465 (98.8)	NA	19	14 (11 covered by RUSP)	1.3
Metabolomics (plasma) (n = 1807 families)	2000	1059 (58.6)	1665 (92.1)	1464 (81)	128	70 (21 covered by RUSP)	7.1

Abbreviations: IEMs, inborn errors of metabolism; NA, not available; RUSP, Recommended Uniform Screening Panel.

^a^ A total of 158 families appeared in both cohorts with 20 of 158 (12.7%) receiving a diagnosis; however, 7 of 158 (4.4%) were positively screened by both testing approaches, whereas 13 of 158 (8.2%) were identified only by metabolomics.

#### Traditional Metabolic Screening Findings

By following a multistep workflow pipeline for data integration and analysis (Figure 1A and as described in the Methods), 159 of 1483 families (10.7%) with biochemical abnormalities were identified. Further integrated analysis revealed that 19 of 159 cases (11.9%) were considered diagnostic for an IEM, giving a final positive diagnostic rate of 1.3% (19 of 1483 cases), whereas an IEM was ruled out for 71 cases (44.7%). The remaining 69 cases (43.4%) lacked other available testing information to confirm the diagnosis. In total, 14 IEMs were identified (Table 1 and eTable 2 in the Supplement). With the exception of L-2/D-2-hydroxyglutaric aciduria, homocystinuria megaloblastic anemia, cblG type, and ethylmalonic encephalopathy, 11 of 14 conditions (78.6%) are included on the RUSP, including 7 conditions covered by the core conditions and 4 disorders covered by RUSP secondary conditions, corresponding to a diagnostic rate of 0.2% for non-RUSP IEMs (3 of 1483 IEMs).

### Metabolomic Screening for IEMs

#### Demographic Characteristics of Clinical Cases

A global meta-analysis process was applied to delineate the findings from plasma metabolomic profiling of 2000 consecutive clinical samples from 1807 unrelated families tested in a similar period as traditional metabolic screening (Table 1). Laboratory sample submission requirements included a detailed clinical note and laboratory test history; however, this guideline was followed only approximately 75% of the time, limiting the analysis of data. In this cohort, most patients were pediatric (1665 patients [92.1%]; mean [SD] age, 8.1 [10.4] years; range, 0-80 years) and male (1059 patients [58.6%]), including 894 children younger than 5 years (49.5%), 771 children and adolescents aged 5 to 21 years (42.6%), and 142 individuals older than 21 years (7.9%). In this cohort, 1464 patients (81.0%) presented with autism spectrum disorder, speech delay, hypotonia, and/or seizures, whereas 219 (12.1%) had nonneurological presentations. Clinical data were not provided for the remaining 124 (6.9%) patients.

#### Overview of Metabolomic Screening

In each individual plasma sample, a mean (SD) of 776 (130) metabolites were detected, and 620 (78) metabolites were *z*-scored. An integrated analysis of metabolomic data considered all available clinical and genetic information for every patient and followed a step-by-step filtering workflow pipeline (Figure 1B). A total of 1807 unique families were stratified according to the metabolic abnormalities revealed by plasma metabolomic profiling into 2 major categories: 912 families (50.5%) with substantial alterations of disease-associated analytes and 895 families (49.5%) with normal or not substantially abnormal profiles.

Among the 912 families with abnormal results, biochemical patterns were categorized as follows: (1) specific and diagnostic for an IEM (128 of 912 families [14.0%]); (2) suggestive of a single IEM or single category of IEM but not definitive (655 of 912 families [71.8%]); or (3) abnormal but nonspecific for a known disease of category (129 of 912 families [14.1%]). Among the 128 families in the diagnostic category, 84 (65.6%) were further confirmed as positive according to additional testing, and 39 different metabolic conditions were identified. The remaining 44 families (34.4%) did not have additional testing available for absolute confirmation of diagnosis and were considered diagnostic on the basis of the metabolomic profile; however, according to our strict assessment criteria, these cases were not included in the final number of confirmed cases reported here.

Details of positive diagnosed cases and known and novel disease-related secondary analytes revealed by metabolomics^21,22,23,24,25,26,27,28,29,30,31,32,33,34,35^ are reported in Table 2 and eTable 3 in the Supplement. Among the 655 families in the suggestive category (Figure 1B), 40 families (6.1%) were confirmed by further testing, including 36 families (90.0%) confirmed via molecular testing. In 53 families (8.1%), a suspected diagnosis was ruled out, and the remaining 562 families (85.8%) were not analyzed because of the lack of available information. This analysis identified 36 different conditions in this category (eTable 3 in the Supplement).

**Table 2.** IEMs Identified by Plasma Clinical Metabolomic Screening

RUSP category and IEM No.	IEM name	OMIM No.^a^	Family No.	Key disease-related analytes detected in plasma^b^
Conditions not on RUSP				
1	Adenylosuccinase deficiency (ADSLD)^23^	103050	28, 85, 390, 499, 584, 1749	N6-succinyladenosine
2	AICA-ribosiduria due to ATIC deficiency	608688	1797	N6-succinyladenosine
3	α-methylacyl-CoA racemase deficiency (AMACRD)^c^	614307	847	Phytanate, 7-α-hydroxy-3-oxo-4-cholestenoate^d^
4	Aromatic L-amino acid decarboxylase deficiency (AADCD)^18,24^	608643	180, 812	3-Methoxytyrosine, 3-methoxytyramine sulfate ↓,^d^ vanillylmandelate ↓, dopamine 3-O-sulfate ↓^d^
5	Autism, susceptibility to, X-linked 6 (AUTSX6)	300872	576, 814, 1461	N6,N6,N6-trimethyllysine, acetylcarnitine ↓, propionylcarnitine ↓, carnitine ↓, deoxycarnitine ↓
6	β-ureidopropionase deficiency (UPB1D)	613161	1258	3-Ureidopropionate, 5,6-dihydrothymine, 5,6-dihydrouracil, 3-aminoisobutyrate ↓
7	Brown-Vialetto-Van Laere syndrome 2 (BVVLS2)^21^	614707	1757	Riboflavin (due to initiation of supplement), pyridoxine and pyridoxate (normalized in response to riboflavin), medium chain (C6, C8, C10:1) and very long chain acylcarnitines (C24), 2-hydroxyglutarate, methylsuccinate, ethylmalonate, kynurenine,^d^ N-formylanthranilic acid,^d^ kynurenate ↓,^d^ picolinate ↓,^d^ methionine sulfone ↓^d^
8	Carbamoyl phosphate synthetase I deficiency, hyperammonemia due to	237300	184	Pyroglutamine,^d^ citrulline ↓
9	Cerebral creatine deficiency syndrome 2 (CCDS2)	612736	57, 234, 347, 1550	Guanidinoacetate, creatine ↓
10	D-bifunctional protein deficiency^33^	261515	958, 1486	1-Lignoceroyl-GPC (24:0), docosadienoate, multiple sphingomyelins ↓, phosphatidylcholines ↓
11	Developmental and epileptic encephalopathy 25, with amelogenesis imperfecta (DEE25)^26^	615905	39, 41, 52	Citrate
12	Dihydrolipoamide dehydrogenase deficiency (DLDD)	246900	137	Lactate, pyruvate, 3-methyl-2-oxobutyrate,^d^ 3-methyl-2-oxovalerate,^d^ 4-methyl-2-oxopentanoate,^d^ 2-hydroxyglutarate
13	Encephalopathy, ethylmalonic (EE)^25^	602473	430, 1246, 1470, 1603	Ethylmalonate, butyrylcarnitine, isobutyrylcarnitine, isovalerylcarnitine, glutarylcarnitine, 2-methylbutyrylcarnitine, methylsuccinate, phenol sulfate ↓, 3-indoxyl sulfate ↓, glycolithocholate sulfate ↓
14	Epilepsy, pyridoxine-dependent (EPD)	266100	102, 933	Pipecolate, 6-oxopiperidine-2-carboxylate
15	Fructose intolerance, hereditary (HFI)	229600	902	Fructose
16	GABA-transaminase deficiency^20^	613163	27, 292, 387, 1333	2-Pyrrolidinone,^d^ succinimide,^d^ succinamic acid^d^
17	Glutaric aciduria III (GA3)	231690	207	Glutarate, arachidate (20:0),^d^ octadecadienedioate (C18:2-DC),^d^ hexadecanedioate,^d^ octadecenedioate (C18:1-DC),^d^ ximenoylcarnitine (C26:1)^d^
18	Glycerol kinase deficiency (GKD)^27^	307030	562, 987	Glycerol, long chain and very long chain monoacylglycerols^d^
19	Glycine encephalopathy (GCE)	605899	514	Glycine
20	Glycogen storage disease 1A (GSD1A)	232200	1347	Lactate, urate, pyruvate, palmitoyl-linoleoyl-glycerol,^d^ glucose ↓
21	Homocystinuria due to deficiency of n(5,10)-methylenetetrahydrofolate reductase activity	236250	156	Methionine sulfoxide ↓,^d^ betaine ↓, methionine ↓, methionine sulfone ↓^d^
22	Homocystinuria-megaloblastic anemia, cblG complementation type (HMAG)	250940	923	S-adenosylhomocysteine,^d^ methionine ↓
23	Hyperornithinemia-hyperammonemia-homocitrullinuria syndrome (HHHS)	238970	557	Ornithine, homocitrulline, N-δ-acetylornithine^d^
24	Isopentenyl-diphosphate delta isomerase 1 (IDI1)^a^	604055	1733	Deoxycholate,^d^ 1-(1-enyl-stearoyl)-2-docosahexaenoyl-GPE (P-18:0/22:6),^d^ octadecadienedioate (C18:2-DC),^d^ dodecadienoate (12:2),^d^ 1-(1-enyl-palmitoyl)-2-linoleoyl-GPE (P-16:0/18:2),^d^ 1-(1-enyl-stearoyl)-2-linoleoyl-GPE (P-18:0/18:2),^d^ 1,2-dilinoleoyl-GPC (18:2/18:2),^d^ multiple sphingomyelins ↓
25	L-2-hydroxyglutaric aciduria (L2HGA)	236792	469	2-Hydroxyglutaric acid
26	Lesch-Nyhan syndrome (LNS)	300322	358	Inosine, uracil
27	Lipoyltransferase 1 deficiency (LIPT1D)^28^	616299	731	Leucine, isoleucine, valine, 2-hydroxyadipate, isovalerylcarnitine, glycine isovalerylglycine, isovalerylcarnitine (C5), 1-stearoyl-2-arachidonoyl-GPI (18:0/20:4), 3-hydroxy-3-methylglutarate, 2-aminoadipate, tyrosine, arginine, serine oxalate (ethanedioate) ↓, malonate ↓
28	Liver failure, infantile, transient (LFIT)	613070	595	3-(4-Hydroxyphenyl) lactate,^d^ phenyllactate,^d^ 4-hydroxyphenylpyruvate,^d^ N-acetylphenylalanine,^d^ fumarate,^d^ alanine,^d^ lactate, pyruvate, glycochenodeoxycholate,^d^ glycohycholate,^d^ taurocholate,^d^ bilirubin
29	Lysinuric protein intolerance (LPI)	222700	1302	Ornithine ↓, urea ↓, arginine ↓, lysine ↓, dimethylarginine ↓^d^
30	Mitochondrial complex V (ATP synthase) deficiency, nuclear type 2 (MC5DN2)	614052	34, 1556	3-Methylglutaconate, 3-methylglutarylcarnitine, alanine, lactate
31	Mitochondrial DNA depletion syndrome 1 (mitochondrial neurogastrointestinal encephalomyopathy type) (MTDPS1)^29^	603041	1120	Thymidine, thymine, 5,6-dihydrothymine ↓
32	Mitochondrial DNA depletion syndrome 9 (encephalomyopathic type with methylmalonic aciduria) (MTDPS9)	245400	50	Succinylcarnitine, propionylcarnitine, methylmalonate, hexanoylcarnitine, butyrylcarnitine, malate ↓
33	Mitochondrial short-chain enoyl-CoA hydratase 1 deficiency (ECHS1D)^30^	616277	1261	β-hydroxyisovalerate, 1-lignoceroyl-GPC (24:0),^d^ 3-hydroxy-3-methylglutarate, laurate (12:0)^d^
34	Multiple mitochondrial dysfunctions syndrome 1 (MMDS1)^31^	605711	1469	Glycine^e^
35	Neurodegeneration, infantile-onset, biotin-responsive (NERIB)	618973	171	Pantothenate (vitamin B5) ↓,^d^ carnitine ↓,^d^ multiple carnitine derivatives ↓^d^
36	Ornithine transcarbamylase deficiency, hyperammonemia due to^32^	311250	177, 819, 1094	Orotate, ornithine, N-carbamoylaspartate,^d^ uridine, uracil, alanine, aspartate, glutamine, citrulline ↓, arginine ↓
37	Peroxisome biogenesis disorder 1A (Zellweger) (PDB1A)^33^	214100	361	Pipecolate, docosadioate,^d^ multiple sphingomyelins ↓
38	Peroxisome biogenesis disorder 4A (Zellweger) (PDB4A)	614862	440	Pipecolate, 1-lignoceryl-GPC,^d^ 7-HOCA,^d^ hexadecanedioate,^d^ octadecanedioate,^d^ eicosanodioate,^d^ docosadioate,^d^ phytanate, multiple sphingomyelins ↓,^d^ plasmalogens ↓, phosphatidylcholines ↓,^d^ phosphatidylethanolamines ↓^d^
39	Peroxisome biogenesis disorder 8A (Zellweger) (PDB8A)	614876	252	Pipecolate, phytanate, long chain fatty acids, 1-lignoceryl-GPC,^d^ 7-HOCA,^d^ hexadecanedioate,^d^ octadecanedioate,^d^ eicosanodioate,^d^ docosadioate,^d^ phytanate, multiple sphingomyelins ↓,^d^ plasmalogens ↓, phosphatidylcholines ↓,^d^ phosphatidylethanolamines ↓^d^
40	Phosphoglycerate dehydrogenase deficiency (PSATD)^19^	610992	1253	Serine ↓, glycine ↓, glycerophosphocholine ↓,^d^ glycerophosphoethanolamine ↓,^d^ multiple lipids ↓^d^
41	Phosphoserine aminotransferase deficiency (PHGDHD)^19^	601815	863, 1063	Serine ↓, glycine ↓, glycerophosphocholine ↓,^d^ glycerophosphoethanolamine ↓,^d^ multiple lipids ↓^d^
42	Pyruvate dehydrogenase, α-1 (PDHA1)	300502	393	Lactate, pyruvate, alanine
43	Short stature, developmental delay, and congenital heart defects (SDDHD; TKT)^34^	617044	1034	Ribitol, arabitol/xylitol, ribonate, erythronate, arabonate/xylonate,^d^ erythritol, ribose
44	Smith-Lemli-Opitz syndrome (SLOS)	270400	373, 647	7-Dehydrocholesterol, cholesterol ↓
45	Spastic paraplegia 9B, autosomal recessive (SPG9B)	616586	60, 1449	Proline ↓, ornithine ↓, citrulline ↓
46	Spondyloepimetaphyseal dysplasia, Genevieve type (SEMDG)	610442	1483	N-acetylglucosamine/N-acetylgalactosamine,^d^ N-acetylneuraminate ↓
47	Succinic semialdehyde dehydrogenase deficiency (SSADHD)	271980	721	2-Pyrrolidinone,^d^ 4-guanidinobutanoate^d^
48	Transaldolase deficiency (TALDOD)^34^	606003	420, 1028	Ribitol, ribonate, erythronate, arabitol/xylitol, erythritol, sedoheptulose
49	Urocanase deficiency (UROCD)^35^	276880	606, 1411	Trans-urocanate, imidazole propionate,^d^ cis-urocanate
RUSP core conditions				
50	Acyl-CoA dehydrogenase, medium-chain, deficiency of (ACADMD)	201450	226, 1441, 1602, 1800	Hexanoylcarnitine, octanoylcarnitine, hexanoylglycine, cis-4-decenoylcarnitine
51	Adrenoleukodystrophy (ALD)	300100	341	Numerous significant abnormalities in bile acid, fatty acid, and lipid metabolism
52	Carnitine deficiency, systemic primary (CDSP)	212140	704, 1222	Carnitine ↓, multiple acylcarnitines ↓
53	Citrullinemia, classic^32^	215700	1006	Citrulline, N-acetylcitrulline^d^
54	Galactosemia I (GALAC1)	230400	1489, 1519	Galactitol, galactonate
55	Glutaric acidemia I (GA1)	231670	916, 1091	Glutarylcarnitine, glutarate
56	HSD10 mitochondrial disease (HSD10MD)	300438	854, 1072, 1450	Tiglylcarnitine, tiglylglycine (rare), 2-hydroxy-3-methylvalerate, β-hydroxyisovalerate, isoleucine, 3-hydroxyisobutyrate, 3-hydroxy-2-ethylpropionate,^d^ 2-methylbutyroylcarnitine^d^
57	Isovaleric acidemia (IVA)	243500	1035	Isovalerylcarnitine, 2-methylbutyroylcarnitine, isovalerylglycine, isovalerate
58	Maple syrup urine disease (MSUD)	248600	86, 1648	Leucine, valine, isoleucine, allo-isoleucine, 2-hydroxy-3-methylvalerate, 3-methyl-2-oxovalerate, isovalerate, β-hydroxyisovalerate, N-acetylisoleucine,^d^ N-acetylleucine,^d^ 3-methyl-2-oxobutyrate^d^
59	Methylmalonic aciduria due to methylmalonyl-CoA mutase deficiency	251000	62, 792, 971, 1121, 1178	Propionylcarnitine, 2-methylcitrate, methylmalonate
60	Phenylketonuria (PKU)	261600	482, 672, 1191, 1299	Phenylalanine, phenylpyruvate, phenyllactate, phenylacetate, N-acetylphenylalanine,^d^ N-formylphenylalanine,^d^ 3-(4-hydroxyphenyl) lactate^d^
61	Propionic acidemia	606054	51, 127, 223, 1223	Propionylcarnitine, propionylglycine, 2-methylcitrate, glycine
62	Tyrosinemia, type I (TYRSN1)	276700	791	3-(4-Hydroxyphenyl) lactate,^d^ 4-hydroxyphenylpyruvate, phenyllactate, tyrosine, N-acetyltyrosine
RUSP secondary conditions				
63	2-Methylbutyryl-CoA dehydrogenase deficiency	610006	1274	2-Methylbutyroylcarnitine, 2-methylbutyrylglycine, 3-hydroxy-2-ethylpropionate, tiglylcarnitine, isobutyrylglycine
64	3-Hydroxyisobutyryl-CoA hydrolase deficiency (HIBCHD)	250620	70	3-Hydroxyisobutyrate ↓
65	Acyl-CoA dehydrogenase, short-chain, deficiency of (ACADSD)	201470	253, 833	Ethylmalonate, butyrylcarnitine, methylsuccinate
66	Argininemia^32^	207800	33, 254, 539, 650, 829, 1683	Arginine, argininate, N-acetylarginine, 2-oxoarginine, dimethylarginine, 4-guanidinobutanoate, homoarginine, orotate, uracil, urea ↓, ornithine ↓
67	Citrullinemia, type II, neonatal onset	605814	1753	Citrulline, homocitrulline,^d^ arginine, argininate,^d^ homoarginine,^d^ methionine, phenyllactate, 4-hydroxyphenylpyruvate, N-acetylphenylalanine,^d^ argininosuccinate, bilirubin, galactonate, galactitol, cholesterol
68	Hyperphenylalaninemia, nonphenylketonuria mild, included	261600	201	Phenylalanine, phenylpyruvate, phenyllactate
69	Methylmalonic aciduria and homocystinuria, cblC type (MAHCC)	277400	315	Methylmalonate, 3-hydroxy-3-methylglutaric acid, propionylcarnitine, methionine ↓
70	Multiple acyl-CoA dehydrogenase deficiency (MADD)	231680	522, 1156, 1532	α-hydroxyisovalerate,^d^ 2-hydroxy-3-methylvalerate, 2-hydroxyglutarate, 2-methylbutyrylglycine, glutarate, glutarylcarnitine, ethylmalonate, methylsuccinate,^d^ butyrylcarnitine and a wide variety of various acylcarnitines

Abbreviations: ↓, Decrease in analyte; IEMs, inborn errors of metabolism; OMIM, Online Mendelian Inheritance in Man; RUSP, Recommended Uniform Screening Panel.

^a^ Mendelian Inheritance in Man numbers are shown for all disorders except isopentenyl-diphosphate-δ-isomerase 1 deficiency, for which the Mendelian Inheritance in Man gene number is provided (https://www.omim.org).

^b^ Analytes listed achieved a *z* score less than or equal to −2 or greater than or equal to 2 compared with reference control population or were identified as a rare molecule and were not *z* scored. All analytes listed represent elevations unless indicated with a downward arrow (↓).

^c^ Pristanate was not identified in this patient sample because of the absence of pristanate in the compound library at the early time of testing; the current platform identifies this metabolite as a rare compound.

^d^ Indicates analytes that were not previously reported for the indicated IEMs and may represent new biomarkers.

^e^ Elevated glycine and lactate were found in cerebrospinal fluid metabolomics.

For the remaining 129 families in the nonspecific category, the inclusion of sequencing data, as well as other genetic information, led to confirmed diagnoses in 3 families (2.3%) with identification of 3 different conditions and 1 non-IEM condition in 1 family (0.78%), whereas suspected diagnoses were ruled out for 5 families (3.9%) (Table 2 and eTable 3 in the Supplement). The remaining 120 families (93.0%) were not further analyzed because of a lack of information.

In total, our analysis confirmed 128 of 912 cases (14.0%) as positive, including 95 cases that were confirmed by molecular testing and 67 cases that had confirmation with additional targeted quantitative metabolic testing, further supporting the reliability of metabolomic profiling. A conservative overall diagnostic rate of 7.1% (approximately 6-fold higher than that for traditional screening) was determined in our cohort of 1807 families.

#### Metabolomic Profiling Expands Diagnostic Screening for IEMs

**Figure 2.**
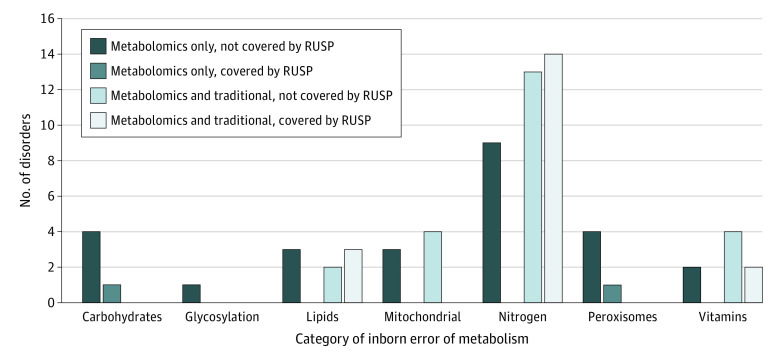
Comparison of Metabolic Conditions Screened by Plasma Clinical Metabolomics, Traditional Metabolic Screening, and Newborn Screening (NBS) In this cohort analysis, clinical metabolomics (plasma) identified 70 metabolic conditions that were categorized into 7 disease groups according to their respective biochemical pathways or disease groups, as well as common pathophysiological mechanisms. The Recommended Uniform Screening Panel (RUSP) was applied as the criterion for determining the conditions screened by NBS. IEMbase version 2.0.0 was applied as the criterion for categorizing the conditions screened by clinical metabolomics and the RUSP. Carbohydrates refers to disorders of carbohydrates, glycosylation refers to congenital disorders of glycosylation, lipids refers to disorders of lipids, mitochondrial refers to mitochondrial disorders of energy metabolism, nitrogen refers to disorders of nitrogen-containing compounds, peroxisomal refers to disorders of peroxisomes and oxalate, and vitamins refers to disorders of vitamins, cofactors, and minerals.

Through this comprehensive, integrated analysis, we were able to successfully identify either key diagnostic metabolites or related secondary metabolites for 70 distinct IEMs in 128 unique families (Table 2 and eTable 3 in the Supplement). Although 21 of these conditions are on the RUSP, including 13 disorders covered as core conditions and 8 as secondary conditions, 49 identified IEMs (70.0%) are not presently included on the RUSP and represent a wide variety of metabolic disorders, including amino acidopathies, organic acidemias, fatty acid oxidation disorders, vitamin or cofactor deficiencies, carbohydrate metabolism disorders, mitochondrial respiratory chain defects, peroxisomal disorders, nucleic acid metabolism disorders, and neurotransmitter abnormalities; 26 of these 49 conditions are not covered by the traditional screening approach, and 7 of these 26 disorders are currently treatable (Figure 2, Table 2, and eTable 4 in the Supplement).

## Discussion

In this cross-sectional study, we conducted a comprehensive comparison of metabolomic screening with traditional metabolic screening to provide insight into the utility and efficacy of both approaches in the identification and diagnosis of inherited metabolic disorders. Traditional combined metabolic screening assays (PAA, ACP, and UOA) had a positive diagnostic rate of 1.3% and identified 14 IEMs, 11 of which (78.6%) are included on the RUSP, identifying only 3 conditions not on the recommended NBS panel. In comparison, the diagnostic rate for clinical metabolomic screening was 7.1%, and the platform successfully identified 21 conditions included in the RUSP, as well as 49 conditions not included in the RUSP (Table 2). Importantly, 26 of these 49 conditions are not covered by the traditional screening approach, and 7 of these 26 disorders are currently treatable, indicating that early screening of these conditions by clinical metabolomics may prevent severe clinical consequences caused by these disorders (eTable 4 in the Supplement). These findings suggest that untargeted metabolomic profiling has a higher diagnostic rate (approximately 6-fold higher) in screening for IEMs and, more importantly, screens for more metabolic diseases compared with the traditional screening approach, supporting the utility of clinical metabolomics as a first-line screening tool for IEMs (Figure 2 and eTable 4 in the Supplement).^7,8,9,36^

We observed that 84 of 128 cases in the diagnostic category were confirmed as positive by integrated analysis with additional genomic and/or biochemical testing, confirming the diagnosis of 39 different metabolic conditions in this category (eTable 3 in the Supplement). These findings suggest that in addition to broadly screening for IEMs, metabolomics alone has the ability to be used as a diagnostic tool for these disorders. For many cases in the suggestive category, although metabolomic profiling indicated abnormalities in specific metabolic pathways, the biochemical profile alone could not distinguish between related disorders. However, subsequent testing, such as DNA sequencing and enzyme analysis, indeed confirmed a specific IEM. In these cases, although the associated metabolomic patterns were not pathognomonic, screening results provided further guidance for follow-up testing, thus preventing incorrect and/or delayed diagnoses in these cases. Of note, a large number of cases lacked further testing results for us to consider in our diagnostic pipeline; therefore, the 7.1% diagnostic rate for clinical metabolomics may be an underestimate.

A major advantage of untargeted metabolomic analysis over targeted metabolic testing is the wide range of metabolites and classes of metabolites that may be assayed in a single biological sample. Similar metabolite coverage by targeted approaches would otherwise require multiple individual clinical tests, which may still focus on only a limited set of analytes from a single biochemical class. Moreover, because most of the compounds in the reference standard library are not clinically available as targeted assays performed in clinical biochemical genetics laboratories in the US, clinical metabolomics has a greater capacity to find novel and specific biomarkers for known IEMs, as well as discovering new metabolic disorders.

In addition, metabolomics also allows for the detection of pathway-related secondary metabolites often not present in targeted assays. These biochemicals may serve as novel and confirmative biomarkers.^20,37^ For example, in GABA-transaminase deficiency (cases 27, 292, 387, and 1333 in Table 2; eTable 3 in the Supplement), metabolomics identified significantly elevated 2-pyrrolidinone (a lactam cyclization form of GABA), succinimide, and succinamic acid, which are more readily detected than labile GABA, and identified as novel biomarkers for this disorder.^20^ A recent publication^38^ has asserted that classic biomarkers for some disorders could be missed by untargeted metabolomics; however, detection of secondary metabolites may still provide sufficient clues to facilitate the follow-up testing of these IEMs.^18^

In this study, we describe a novel clinical metabolomics method that was applied in the clinical screening and diagnosis of IEMs. Disease identification by metabolomic profiling is based on the substantial alterations of key biomarkers and is buttressed by the pattern of findings within known biochemical pathways. This comprehensive approach is especially helpful when patients present with a nonspecific neurologic phenotype or in cases where prior metabolic testing has been unrevealing. With current technologies, the diagnostic yield of the traditional screening approach is limited beyond the identification of disorders already contained in the RUSP and screened for at birth. However, our results and those of others demonstrate that metabolomics can detect many disorders not included in most NBS panels.^12,13,14,21,39^ Therefore, plasma metabolomic analysis should be considered as an initial screening approach when screening for IEMs, and it may also be useful to provide functional evidence for genomic variants of uncertain significance.^22^

### Limitations

This untargeted metabolomic analysis platform has several limitations. First, the test is designed to detect small molecules and will not detect larger metabolites such as complex oligosaccharides and lipids, potentially limiting the applicability for lysosomal disorders or congenital disorders of glycosylation. Second, some clinically relevant molecules that require special extraction methods (eg, homocysteine) are not identified in this assay. Third, semiquantitative *z* scores, not absolute values, for measured compounds are provided in this screening assay. Fourth, the current turnaround time for this assay is 14 to 21 days, limiting its use for acute metabolic interventions; however, statistical analysis with reporting within 3 of 5 days of sample receipt is possible. Fifth, as with any metabolic testing, diet, medication, or clinical status at the time of sample collection may mask or otherwise alter metabolic abnormalities; thus, a nondiagnostic metabolic assessment does not completely rule out the possibility of underlying metabolic disease.

## Conclusions

In this cross-sectional study, we presented a clinical untargeted metabolomic profiling method with a higher diagnostic screening rate for IEMs (approximately 6-fold higher) that detected more IEMs than the traditional metabolic screening approach, identifying many disorders not included in NBS. With the implementation of expanded NBS, our results support the need to use a broader screening approach, such as untargeted clinical metabolomics or a large panel of targeted analytes, to more comprehensively assess for metabolic disorders in the initial assessment of patients for IEMs and especially for the initial evaluation of neurological phenotypes that include intellectual disability, seizures, global developmental delays, or autism spectrum disorder. Furthermore, in consideration of the increasing use of genome first approaches to diagnosis, including exome or whole genome sequencing, clinical metabolomics offers a broad spectrum functional screen for IEMs that can support a comprehensive approach to genetic diagnosis.

## Notes

Supplement. **eAppendix 1.** Traditional Biochemical Screening **eAppendix 2.** Metabolomic Analyses eReferences **eTable 1.** RUSP Conditions Covered by Traditional Screening and Clinical Metabolomics **eTable 2.** Disorders Identified by Traditional Screening **eTable 3.** Clinical Description of Positively Diagnosed Metabolomic Cohort **eTable 4.** Categorized Metabolic Conditions Screened by Clinical Plasma Metabolomics and Covered by Traditional Screening and/or the RUSP
