# Supplementary material for: Comparison of Untargeted Metabolomic Profiling vs Traditional Metabolic Screening to Identify Inborn Errors of Metabolism
Source: JAMA Netw Open. 2021 Jul 12;4(7):e2114155. doi: 10.1001/jamanetworkopen.2021.14155 (PMC8276086; doi:10.1001/jamanetworkopen.2021.14155)
Supplement: Supplement. — eAppendix 1. Traditional Biochemical Screening eAppendix 2. Metabolomic Analyses eReferences eTable 1. RUSP Conditions Covered by Traditional Screening and Clinical Metabolomics eTable 2. Disorders Identified by Traditional Screening eTable 3. Clinical Description of Positively Diagnosed Metabolomic Cohort eTable 4. Categorized Metabolic Conditions Screened by Clinical Plasma Metabolomics and Covered by Traditional Screening and/or the RUSP [file jamanetwopen-e2114155-s001.pdf]

Supplemental Online Content

Liu N, Xiao J, Gijavanekar C, et al. Comparison of untargeted metabolomic profiling vs traditional metabolic screening to identify inborn errors of metabolism. *JAMA Netw Open*. 2021;4(7):e2114155. doi:10.1001/jamanetworkopen.2021.14155

eAppendix 1. Traditional Biochemical Screening
eAppendix 2. Metabolomic Analyses
eReferences
eTable 1. RUSP Conditions Covered by Traditional Screening and Clinical Metabolomics
eTable 2. Disorders Identified by Traditional Screening
eTable 3. Clinical Description of Positively Diagnosed Metabolomic Cohort
eTable 4. Categorized Metabolic Conditions Screened by Clinical Plasma Metabolomics and Covered by Traditional Screening and/or the RUSP

This supplemental material has been provided by the authors to give readers additional information about their work.

© 2021 Liu N et al. *JAMA Network Open*.

eAppendix 1. Traditional Biochemical Screening

All clinical testing was performed at Baylor Genetics using standard clinical biochemical testing protocols.

For quantitative amino acid analysis, plasma samples were collected and kept frozen until analysis. Specimens were deproteinized by mixing with Seraprep (Pickering Laboratory) with internal standard in 1:1 ratio and diluted with Biochrom loading buffer. After centrifugation and filtration, the supernatants were analyzed using cation exchange chromatography with post-column ninhydrin derivatization on either a Hitachi L-8900 or Biochrom 30 amino acid analyzer.[1]

For quantitative acylcarnitine profiles (ACP), plasma samples were collected and kept frozen until analysis. Thawed samples were mixed with internal standard (Cambridge Isotope Laboratories) resuspended in 100% methanol, deproteinized and centrifuged. The supernatant is dried under N_2 and subsequently derivatized in butanol-HCl. After drying, acylcarnitines were resuspended in 100% methanol and applied to analyzed in an Acquity TQ tandem MS (Waters).[2]

Urine organic acids (UOA) were isolated and qualitative analysis was performed as previously described.[3] The urine specimens were collected and stored frozen until analysis. Samples were acidified to pH = 1 using 6 N hydrochloric acid (J.T. Baker), and the urine organic acids were extracted by applying to a Chem Elute extraction column (Agilent Technologies) and eluted into ether organic solvents, dried, derivatized with Regisil (Regis Technologies, Inc.), and subsequently analyzed using gas chromatography coupled MS (Agilent). Compounds were identified based on both elution time and fragmentation spectrum.

eAppendix 2. Metabolomic Analyses

Clinical metabolomic analyses were performed on plasma samples as previously described.[4-6] Briefly, small molecules ranging from 75-1000 Da were extracted from plasma derived from an EDTA whole blood sample using an 80% methanol solution containing four extraction efficiency standards (tridecanoic acid, 4-Cl-phenylalanine, 2-flurophenylglycine, and d6-cholesterol). The clarified supernatant was analyzed by one of two different platform configurations, depending on the date the sample was received in the laboratory.

The first platform configuration consisted of four chromatographic analyses: GC-MS, LC-MS/MS in positive mode (LCMS Pos), LC-MS/MS in negative mode (LCMS Neg), and a LC-MS/MS Polar method (LCMS Pol). GC-MS was performed on bistrimethyl-silyl-trifluoroacetamide derivatized analytes using a Trace DSQ fast-scanning single-quadrupole mass spectrometer (Thermo-Finnigan). For LC/MS Neg and LCMS Pos methods, chromatographic separation was completed using an ACQUITY UPLC (Waters) equipped with a Waters BEH C18 column followed by analysis with an Orbitrap Elite high-resolution mass spectrometer (Thermo-Finnigan). This protocol was followed for samples received prior to May 2015.

The second configuration (for samples received after May 2015) utilized the same mass spectrometry methods but utilized the following chromatographic methods: LCMS Neg (same as the previous configuration), LCMS Pol (same as previous configuration), LCMS positive ion method focusing on lipophilic compounds (LCMS Pos Lipid), and LCMS positive ion method focusing on polar compounds (LCMS Pos Polar).[4,7,8]

Metabolites were identified by matching the ion's chromatographic retention index, accurate mass, and mass spectral fragmentation signatures with a LC-MS compound reference library consisting of >3,000 entries created from authentic standards.[9] To compare patient sample data to the control reference population, biochemical raw spectral intensity values were normalized to the compound median of the anchor samples and then z-scored using the associated mean and standard deviation of the anchor median-normalized reference population. Z-scores represent the standard deviation of a compound in the patient sample away from the reference population mean. Analytes with a z-score < -2 or > +2 represent significantly altered compounds.[5]

© 2021 Liu N et al. *JAMA Network Open.*

eReferences

1. Walker V, Mills GA. Quantitative methods for amino acid analysis in biological fluids. *Ann Clin Biochem.* 1995;32 (Pt 1):28-57. doi: 10.1177/000456329503200103
2. Vreken P, van Lint AE, Bootsma AH, Overmars H, Wanders RJ, van Gennip AH. Quantitative plasma acylcarnitine analysis using electrospray tandem mass spectrometry for the diagnosis of organic acidaemias and fatty acid oxidation defects. *J Inherit Metab Dis.* 1999;22(3):302-306. doi: 10.1023/a:1005587617745
3. The Metabolic and Molecular Bases of Inherited Disease (Scriver, C. R., Beaudet, A. L., Sly, W. S., Valle, D., Childs, B., Kinzler, K. W., and Vogelstein, B., eds., 8th ed., McGraw-Hill, New-York, 2001, 7012 p., $550.00). *Biochemistry (Moscow).* 2002;67(5):611-612. doi: 10.1023/A:1017418800320
4. Kennedy AD, Wittmann BM, Evans AM, et al. Metabolomics in the clinic: A review of the shared and unique features of untargeted metabolomics for clinical research and clinical testing. *J Mass Spectrom.* 2018;53(11):1143-1154. doi: 10.1002/jms.4292
5. Ford L, Kennedy AD, Goodman KD, et al. Precision of a Clinical Metabolomics Profiling Platform for Use in the Identification of Inborn Errors of Metabolism. *J Appl Lab Med.* 2020;5(2):342-356. doi: 10.1093/jalm/jfz026
6. Miller MJ, Kennedy AD, Eckhart AD, et al. Untargeted metabolomic analysis for the clinical screening of inborn errors of metabolism. *J Inherit Metab Dis.* 2015;38(6):1029-1039. doi: 10.1007/s10545-015-9843-7
7. Kennedy AD, Pappan KL, Donti T, et al. 2-Pyrrolidinone and Succinimide as Clinical Screening Biomarkers for GABA-Transaminase Deficiency: Anti-seizure Medications Impact Accurate Diagnosis. *Front Neurosci.* 2019;13:394. doi: 10.3389/fnins.2019.00394
8. Cappuccio G, Pinelli M, Alagia M, et al. Biochemical phenotyping unravels novel metabolic abnormalities and potential biomarkers associated with treatment of GLUT1 deficiency with ketogenic diet. *PLoS One.* 2017;12(9):e0184022.
9. Dehaven CD, Evans AM, Dai H, Lawton KA. Organization of GC/MS and LC/MS metabolomics data into chemical libraries. *J Cheminform.* 2010;2(1):9. doi: 10.1371/journal.pone.0184022

© 2021 Liu N et al. *JAMA Network Open*.

eTable 1. RUSP Conditions Covered by Traditional Screening and Clinical Metabolomics[a]

Group 1. RUSP 25 Core Conditions Covered by Traditional Screening and Clinical Metabolomics				
	**IEM (MIM #)**	**Covered by Metabolomics & Traditional (N=21)**	**Covered by Metabolomics Only (N=2)**	**Covered by Traditional Only (N=0)**
1	Propionic acidemia (606054)	Yes		
2	Methylmalonic aciduria due to methylmalonyl-CoA mutase deficiency (251000)	Yes		
3	Methylmalonic acidemia (cobalamin disorders) (251100; 277410; 608419)	Yes		
4	Isovaleric acidemia; IVA (243500)	Yes		
5	3-Methylcrotonyl-CoA carboxylase deficiency (210200; 210210)	Yes		
6	3-hydroxy-3-methylglutaryl-CoA lyase deficiency; HMGCLD (246450)	Yes		
7	Holocarboxylase synthetase deficiency (253270)	Yes		
8	Acetyl-CoA acetyltransferase 1; ACAT1 (607809)	Yes		
9	Glutaric acidemia I; GA1 (231670)	Yes		
10	Carnitine deficiency, systemic primary; CDSP (212140)	Yes		
11	Acyl-CoA dehydrogenase, medium-chain, deficiency of; ACADMD (201450)	Yes		
12	Acyl-CoA dehydrogenase, very long-chain, deficiency of; ACADVL (201475)	Yes		
13	Long-chain 3-hydroxyacyl-CoA dehydrogenase deficiency (609016)	Yes		
14	Mitochondrial trifunctional protein deficiency; MTPD (609015)	Yes		
15	Argininosuccinic aciduria (207900)	Yes		
16	Citrullinemia, classic (215700)	Yes		
17	Maple syrup urine disease; MSUD (248600)	Yes		
18	Homocystinuria due to cystathionine beta-synthase deficiency (236200)	Yes		
19	Phenylketonuria; PKU (261600)	Yes		
20	Tyrosinemia, type I; TYRSN1 (276700)	Yes		
21	Biotinidase deficiency (253260)	Yes		
22	Galactosemia I; GALAC1 (230400)		Yes	
23	Glycogen storage disease type II; GSD2 (232300)			
24	Mucopolysaccharidosis Type 1 (607014; 607016)			
25	Adrenoleukodystrophy; ALD (300100)		Yes	
**Group 2. RUSP 24 Secondary Conditions Covered by Traditional Screening and Clinical Metabolomics**				
	**IEM (MIM #)**	**Covered by Metabolomics & Traditional (N=22)**	**Covered by Metabolomics Only (N=2)**	**Covered by Traditional Only (N=0)**
26	Methylmalonic aciduria and homocystinuria (277400, 277410, 277380, 614857, 309541)	Yes		
27	Malonyl-CoA decarboxylase deficiency (248360)	Yes		

© 2021 Liu N et al. *JAMA Network Open*.

Group 2. RUSP 24 Secondary Conditions Covered by Traditional Screening and Clinical Metabolomics				
	**IEM (MIM #)**	**Covered by Metabolomics & Traditional (N=22)**	**Covered by Metabolomics Only (N=2)**	**Covered by Traditional Only (N=0)**
28	Isobutyryl-CoA dehydrogenase deficiency; IBDD (611283)	Yes		
29	2-methylbutyryl-CoA dehydrogenase deficiency (610006)	Yes		
30	3-Methylglutaconic aciduria (614739, 250950, 616271, 258501, 617698, 617248, 250951, 610198)	Yes		
31	HSD10 mitochondrial disease; HSD10MD (300438)	Yes		
32	Acyl-CoA dehydrogenase, short-chain, deficiency of; ACADSD (201470)	Yes		
33	Medium/short-chain L-3-hydroxyacyl-CoA dehydrogenase deficiency (231530)	Yes		
34	Multiple acyl-CoA dehydrogenase deficiency; MADD (231680)	Yes		
35	Medium-chain ketoacyl-CoA thiolase deficiency (602199)	Yes		
36	2,4-dienoyl-CoA reductase deficiency; DECRD (616034)	Yes		
37	Carnitine palmitoyltransferase I deficiency (255120)	Yes		
38	Carnitine palmitoyltransferase II deficiency (600649, 608836, 255110)	Yes		
39	Carnitine acylcarnitine translocase deficiency; CACTD (212138)	Yes		
40	Argininemia (207800)	Yes		
41	Citrullinemia, type II, neonatal onset (605814)	Yes		
42	Hypermethioninemia (613752, 606664, 250850)	Yes		
43	Hyperphenylalaninemia, non-PKU mild, included (261600)	Yes		
44	Biopterin defect in cofactor biosynthesis (233910, 128230, 261640, 261630)	Yes		
45	Biopterin defect in cofactor regeneration (233910, 264070, 261640, 261630)	Yes		
46	Tyrosinemia, type II; TYRSN2 (276600)	Yes		
47	Tyrosinemia, type III; TYRSN3 (276710)	Yes		
48	Galactosemia III; GALAC3 (230350)		Yes	
49	Galactosemia II; GALAC2 (230200)		Yes	

[a]A complete list of 49 conditions in current RUSP, including 25 core and 24 secondary conditions are listed and whether these conditions are covered by either clinical plasma metabolomics or traditional screening (plasma amino acids, urine organic acids, and plasma acylcarnitine profile) is indicated. Conditions are listed in the order of RUSP.

Abbreviations: RUSP, Recommended Uniform Screening Panel (https://www.hrsa.gov/advisory-committees/heritable-disorders/rusp/index.html).

© 2021 Liu N et al. *JAMA Network Open*.

eTable 2. Disorders Identified by Traditional Screening

	IEM	MIM	Samples (families)	Traditional Screening Findings		
				PAA	ACP	UOA
**Group 1. 3 conditions screened by the traditional screening approach but not covered by RUSP**						
1	L-2-hydroxyglutaric aciduria; L2HGA	236792	1 (1)	-	-	2-hydroxyglutaric acid↑
2	Encephalopathy, ethylmalonic; EE	602473	1 (1)	-	Butyrylcarnitine (C4)↑	Ethylmalonic acid↑<br>Methylsuccinic acid↑<br>Fumaric acid↑<br>Citric acid↑
3	Homocystinuria-megaloblastic anemia, cblG complementation type; HMAG	250940	1 (1)	Methionine↓	Butyrylcarnitine (C4)↑	-
**Group 2. 11 conditions screened by traditional screening and covered by RUSP**						
**Subgroup A. 7 conditions screened by the traditional screening and covered by RUSP core conditions**						
4	Acyl-CoA dehydrogenase, medium-chain, deficiency of; ACADMD	201450	1 (1)	Tyrosine↑<br>Proline↑<br>Ornithine↑	Hexanoylcarnitine (C6)↑<br>Octanoylcarnitine (C8)↑<br>Decenoylcarnitine (C10:1)↑<br>Decanoylcarnitine (C10)↑	Succinic acid↑
5	Citrullinemia, classic	215700	1 (1)	Citrulline↑	Free carnitine↓	Glutaric acid↑<br>5-oxoproline↑
6	Glutaric acidemia I; GA1	231670	1 (1)	Glutamine↑	Glutarylcarnitine (C5DC)↑	Glutaric acid↑
7	Isovaleric acidemia; IVA	243500	1 (1)	-	Pentanoylcarnitine (C5)↑	Isovalerylglycine↑
8	Methylmalonic aciduria, vitamin B12-responsive, cblB type	251110	1 (1)	Glycine↑	Propionylcarnitine (C3)↑	Methylmalonic acid↑<br>Methylcitric acid↑
9	Phenylketonuria; PKU	261600	1 (1)	Phenylalanine↑	-	-
10	Propionic acidemia	606054	3 (3)	Glycine↑	Propionylcarnitine↑	3-hydroxypropionic acid↑<br>Propionylglycine↑<br>TIglylglycine↑<br>Methylcitric acid↑
**Subgroup B. 4 conditions screened by the traditional screening and covered by RUSP secondary conditions**						
11	Argininemia	207800	1 (1)	Arginine↑	Free carnitine↓	-
12	Methylmalonic aciduria and homocystinuria, cblC type; MAHCC	277400	3 (3)	-	Propionylcarnitine (C3)↑	Methylmalonic acid↑
13	Multiple acyl-CoA dehydrogenase deficiency; MADD	231680	2 (2)	-	Numerous acylcarnitines (short, medium and long chain)↑, free carnitine↓	Glutaric acid↑<br>3-hydroxybutyric acid↑<br>Acetoacetic acid↑
14	HSD10 mitochondrial disease; HSD10MD	300438	1 (1)	-	Pentenoylcarnitine (C5:1)↑	Tiglylglycine↑<br>2-methyl-3-OH butyric acid↑<br>Lactic acid↑

Up arrows (↑) and down arrows (↓) indicate analytes with elevated or reduced levels, respectively. (-) indicates normal or no significant abnormalities in the test.
Abbreviations: PAA, plasma amino acids; UOA, urine organic acids; ACP, plasma acylcarnitine profile; RUSP, Recommended Uniform Screening Panel (https://www.hrsa.gov/advisory-committees/heritable-disorders/rusp/index.html).

© 2021 Liu N et al. *JAMA Network Open*.

eTable 3. Clinical Description of Positively Diagnosed Metabolomic Cohort

Family[a] Number	Gender	Age (years)	Phenotype	Neuro[b]	Associated IEM	MIM#	Gene	Zygosity	Inheritance	Gene Accession ID, GRCh 37 (MANE Select)	Nucleotide Change	Amino Acid Change	Canonical Allele Identifier	Variation ID (ClinVar)	Pathogenicity (Clinical reports)	Metabolomic Findings	Other Biochemical Testing Findings
**Group 1. 84 cases with metabolomic abnormalities diagnostic for IEMs and further confirmed by available sequencing tests, biochemical targeted tests, and clinical phenotypes: 39 different metabolic conditions identified**																	
**Subgroup A. 73 cases screened by clinical metabolomics only and not screened by traditional screening approach in this study**																	
27	M	6	Seizures, cortical visual impairment, non-verbal, and non-ambulatory	Yes	GABA-transaminase deficiency	613163	*ABAT*	Hom	AR	NM_020686.6	c.631C>T	NP_065737.2:p.Leu211Phe	CA175085	162034	P	2-pyrrolidinone, succinimide, succinamic acid	PAA:-<br>ACP: C10:1 acylcarnitine
28	F	16	Epilepsy, central hypoventilation and central sleep apnea, hypothyroidism, cyclic vomiting syndrome, dysphagia and narcolepsy, seizure and developmental delay	Yes	Adenylosuccinase deficiency; ADSLD	103050	*ADSL*	Hom	AR	NM_000026.4	c.1339T>C	NP_000017.1:p.Ser447Pro	CA313120	204801	P	N6-succinyladenosine	PAA: arginine[↓], tyrosine[↓]<br>ACP: numerous medium and very long chain acylcarnitines<br>U-PUR: -
85	M	14	Seizures developmental delay	Yes	Adenylosuccinase deficiency; ADSLD	103050	*ADSL*	Het	AR	NM_000026.4	c.1123C>T	NP_000017.1:p.Gln375Ter	CA411646421	450064	LP	N-6 succinyladenosine	Metabolomics-U: N6-succinyladenosine<br>GAMT-P: -<br>PUR-U: succinyladenosine
								Het		NM_000026.4	c.886C>T	NP_000017.1:p.Arg296Trp	CA10247865	450065	VUS		
102	F	7	Epilepsy, pyridoxine-dependent	Yes	Epilepsy, pyridoxine-dependent; EPD	266100	*ALDH7A1*	Hom	AR	NM_001182.5	c.312+1G>A	NP_001173.2:p.=	CA3389815	265384	P	Pipecolate, 6-oxopiperidine-2-carboxylate, pyridoxate (supplement), pyridoxal (supplement)	ACP: C16 and C16:1 acylcarnitines<br>CSFAA: -<br>AASA-P: pipecolic acid
127	M	6	Hyperammonemia and developmental delay	Yes	Propionic acidemia	606054	*PCCB*	Hom	AR	NM_000532.5	c.1172T>C	NP_000523.2:p.Phe391Ser	CA354648766	N/A	P	Propionylcarnitine, propionylglycine, 2-methylcitrate, glycine	Newborn screening: consistent with propionic acidemia<br>PAA: glycine<br>ACP: C3 acylcarnitine
180	M	4	Severe speech and motor delays, generalized brain atrophy with increased ventricular and sulcal spaces, and facial dysmorphism	Yes	Aromatic L-amino acid decarboxylase deficiency	608643	*DDC*	Het	AR	NM_000790.3	c.286G>A	NP_000781.1:p.Gly96Arg	CA367529579	N/A	LP	3-methoxytyrosine, 3-methoxytyramine sulfate [↓], vanillylmandelate [↓], dopamine 3-O-sulfate [↓]	PAA: -<br>UOA: -<br>ACP: -
								Het		NM_000790.3	c.260C>T	NP_000781.1:p.Pro87Leu	CA4262432	N/A	LP		
201	F	3	Delayed motor milestones, delayed speech, developmental regression, hypotonia, seizure disorder, abnormal movements, dysmorphic features, structural brain abnormalities, and eye problems	Yes	Hyperphenylalaninemia, non-PKU mild, included	261600	*PAH*	Het	AR	NM_000277.3	c.842+1G>A	NP_000268.1:p.=	CA229811	599	P	Phenylalanine, phenylpyruvate, phenyllactate	Newborn screening: phenylalanine<br>PAA: phenylalanine
								Het		NM_000277.3	c.805A>C	NP_000268.1:p.Ile269Leu	CA229775	102842	VUS		
226	M	6	Vomiting, stomach problems, nausea, gastroesophageal reflux, possible delayed gastric emptying, chronically low body weight and increased urinary lactate	No	Acyl-CoA dehydrogenase, medium-chain, deficiency of; ACADMD	201450	*ACADM*	Het	AR	NM_000016.6	c.985A>G	NP_000007.1:p.Lys329Glu	CA252821	3586	P	Hexanoylcarnitine, octanoylcarnitine, hexanoylglycine, cis-4-decenoylcarnitine	ACP: C8, C10 and C10:1 acylcarnitines
								Het		NM_000016.6	c.617G>A	NP_000007.1:p.Arg206His	CA220183	92268	P		
234	M	14	Global developmental delay, delayed speech, behavioral problems and autism spectrum disorder	Yes	Cerebral creatine deficiency syndrome 2; CCDS2	612736	*GAMT*	Hom	AR	NM_000156.6	c.79T>C	NP_000147.1:p.Tyr27His	CA295620	167131	VUS	Guanidinoacetate, creatine [↓]	UOA: 3-hydroxybutyric and acetoacetic acids<br>GAMT-P: guanidinoacetate<br>UCT-U: guanidinoacetate/creatinine ratio
253	F	3	Chronic lung disease, congenital heart anomalies (atrial and ventricular septal defects), renal medullary sponge disease, gastro-esophageal reflux, developmental delays, course facial features	Yes	Acyl-CoA dehydrogenase, short-chain, deficiency of; ACADSD	201470	*ACADS*	Hom	AR	NM_000017.4	c.934-5T>A	NP_000008.1:p.=	CA6831144	N/A	VUS	Ethylmalonate, methylsuccinate, butyrylcarnitine	ACP: C4 acylcarnitine
254	F	7	Granulomatous hepatitis of unclear etiology, factor VII deficiency, "febrile seizures" with about four episodes, tip-toe walking, midline stereotypic hand movements, autism spectrum disorder, developmental delay, speech and language delay and myopia	Yes	Argininemia	207800	*ARG1*	Hom	AR	NM_000045.3	c.466-1G>C	NP_000036.2:p.=	CA16618244	419876	P	N-acetylarginine, N-methylproline, 2-oxoarginine, 4-guanidinobutanoate, arginine, homoarginine, urea [↓], ornithine [↓]	PAA: arginine<br>ARG-R: deficient
292	M	1	Hypotonia, developmental delay, constipation, gastroesophageal reflux, abnormal and involuntary movement, and delayed myelination. Facial hemangioma and recurrent ear infections with tympanostomies	Yes	GABA-transaminase deficiency	613163	*ABAT*	Het	AR	NM_020686.6	c.454C>T	NP_065737.2:p.Pro152Ser	CA394688322	635253	VUS	2-pyrrolidinone, succinamic acid, succinimide	UOA: -
								Het		NM_020686.6	c.1393G>C	NP_065737.2:p.Gly465Arg	CA394691458	635254	VUS		
373	M	10	Microcephaly, developmental delays, hypotonia, severe sleep apnea, dystonia, and a complex cardiac defect	Yes	Smith-Lemli-Opitz syndrome; SLOS	270400	*DHCR7*	N.P	AR	N.P	N.P	N.P	N.P	N.P	N.P	7-dehydrocholesterol, cholesterol [↓]	No other tests
387	M	4	Delayed motor milestones, delayed speech, intellectual disability, hypotonia, ataxia, obesity, and strabismus	Yes	GABA-transaminase deficiency	613163	*ABAT*	Het	AR	NM_020686.6	c.168+1G>A	NP_065737.2:p.=	CA394692408	635252	P	2-pyrrolidinone, succinimide, succinamic acid	CSF metabolomics: 2-pyrrolidinone, succinimide, succinamic acid
								Het		NM_020686.6	c.638T>G	NP_065737.2:p.Phe213Cys	CA394688780	635255	VUS		
390	F	2	Developmental delays	Yes	Adenylosuccinase deficiency; ADSLD	103050	*ADSL*	Het	AR	NM_000026.4	c.736A>G	NP_000017.1:p.Lys246Glu	CA115569	2466	P	N-6 succinyladenosine	PUR-U: succinyladenosine
								Het		NM_000026.4	c.947T>C	NP_000017.1:p.Met316Thr	CA324458527	N/A	VUS		
420	M	1	Developmental delays, intellectual disability, liver and renal disease, loose skin	Yes	Transaldolase deficiency; TALDOD	606003	*TALDO1*	Hom	AR	NM_006755.2	c.574C>T	NP_006746.1:p.Arg192Cys	CA5788214	381759	P	Ribose, arabitol/xylitol, erythritol, ribitol, erythronate, ribonate, arabonate/xylonate, sedo heptuloase	POLY-U: erythritol, ribitol, arabitol, sedoheptulose
482	F	25	Migraines, eczema, anxiety, and cyclic vomiting, not on special diet	No	Phenylketonuria; PKU	261600	*PAH*	Hom	AR	NM_000277.3	c.1042C>G	NP_000268.1:p.Leu348Val	CA220576	92727	P	Phenylalanine, phenylpyruvate, phenyllactate, γ-glutamylphenylalanine, phenylalaninylphenylalanine, N-acetylphenylalanine, N-formylphenylalanine	No other tests
499	M	0.5	Developmental delay, infantile spasms with intractable epilepsy	Yes	Adenylosuccinase deficiency; ADSLD	103050	*ADSL*	Hom	AR	NM_000026.4	c.1277G>A	NP_000017.1:p.Arg426His	CA115565	2462	P	N6-succinyladenosine, adenosine	CSF neurotransmitters: succinyladenosine
539	F	0.92	High arginine. Glutamine normal. Clinically doing well on dietary management	No	Argininemia	207800	*ARG1*	Het	AR	NM_000045.3	c.500C>T	NP_000036.2:p.Pro167Leu	CA365651321	N/A	Unclassified	Uracil, N-acetylarginine, 5,6-dihydrouracil, orotate, arginine, homoarginine, uridine, 4-guanidinobutanoate, homocitrulline, glutamine, urea [↓]	ARG-R: deficient
								Het		NM_000045.3	c.533G>A	NP_000036.2:p.Gly178Asp	CA365651438	N/A	Unclassified		
562	M	14	Pseudohypertrigylceridemia, short stature	No	Glycerol kinase deficiency; GKD	307030	*GK*	Hem	XLR	NM_000167.5	c.1357G>A	NP_000158.1:p.Glu453Lys	CA412641787	N/A	Unclassified	Glycerol, 1-arachidonylglycerol (20:4)	No other tests
606	F	5	Intellectual disability and autism spectrum disorder features, with EEG abnormalities and left hippocampal sclerosis	Yes	Urocanase deficiency; UROCD	276880	*UROC1*	Hom	AR	NM_144639.3	c.855G>A	NP_653240.1:p.Trp285Ter	CA2591341	N/A	P	Trans-urocanate, imidazole propionate, cis-urocanate	No other tests
647	M	3	Significant developmental delay, hypotonia and undescended tests, sleep disturbance, gastrointestinal symptoms, and ptosis	Yes	Smith-Lemli-Opitz syndrome; SLOS	270400	*DHCR7*	Het	AR	NM_001360.3	c.841G>A	NP_001351.2:p.Val281Met	CA221680	93724	P	7-dehydrocholesterol, cholesterol [↓], sphingolipids [↓], plasmalogens [↓]	No other tests
								Het		NM_001360.3	c.1210C>T	NP_001351.2:p.Arg404Cys	CA340615	6788	P		
650	M	18	Diplegia, intellectual disability and seizures	Yes	Argininemia	207800	*ARG1*	Het	AR	NM_000045.3	c.295G>A	NP_000036.2:p.Gly99Arg	CA3999210	551529	VUS	Homoarginine, N-acetylarginine, guanidinoacetate, 4-guanidinobutanoate, orotate, arginine, urea [↓], ornithine [↓]	No other tests
								Het		NM_000045.3	c.814G>A	NP_000036.2:p.Gly272Arg	CA365653429	N/A	VUS		
812	F	0.5	Severe hypotonia, delayed motor milestones, feeding difficulties, breathing difficulty, increased congestion, microcephaly and a history of meconium aspirations without respiratory symptoms at birth	Yes	Aromatic L-amino acid decarboxylase deficiency	608643	*DDC*	Hom	AR	NM_001082971.2	c.714+4A>T	NP_001076440.2:p.=	CA4262295	864024	P	3-methoxytyrosine, 3-methoxytyramine [↓]	ACP: C10, C10:1 and C8 acylcarnitines
814	M	7	Partial complex seizures, developmental delay, attention deficit hyperactivity disorder, intention tremor, macrocephaly, frontal bossing, pointed chin, and flat and smooth philtrum	Yes	Autism, susceptibility to, X-linked 6; AUTSX6	300872	*TMLHE*	Hem	XLR	N/A	GRCh37/hg19 arr Xq28(chrX:154773423-154797292)x0	N/A	N/A	N/A	Unclassified	N6,N6,N6-trimethyllysine, acetylcarnitine [↓], propionylcarnitine [↓], carnitine [↓], deoxycarnitine [↓]	No other tests

© 2021 Liu N et al. *JAMA Network Open*.

Family[a] Number	Gender	Age (years)	Phenotype	Neuro[b]	Associated IEM	MIM#	Gene	Zygosity	Inheritance	Gene Accession ID, GRCh 37 (MANE Select)	Nucleotide Change	Amino Acid Change	Canonical Allele Identifier	Variation ID (ClinVar)	Pathogenicity (Clinical reports)	Metabolomic Findings	Other Biochemical Testing Findings
819	M	1	Intermittent and cyclic vomiting, lethargy, elevated transaminases, prolonged INR	No	Ornithine transcarbamylase deficiency, hyperammonemia due to	311250	*OTC*	Hem	XLR	NM_000531.6	c.589G>A	NP_000522.3:p.Gly197Arg	CA224695	97260	P	Orotate, uridine, N-carbamoylaspartate, uracil, 5,6-dihydrouracil, citrulline [↓], arginine [↓], ornithine [↓], guanidinoacetate [↓]	PAA: citrulline [↓]<br>OROT-U: orotate
863	M	4	Global developmental delay, medically refractory epilepsy, congenital cataracts, sensorineural hearing loss, microcephaly, short stature, spasticity, multiple contractures and agenesis of the corpus callosum	Yes	Phosphoglycerate dehydrogenase deficiency; PHGDHD	601815	*PHGDH*	Het	AR	NM_006623.4	c.290+2T>C	NP_006614.2:p.=	CA10602729	280721	P	Serine [↓], glycine [↓], glycerophosphocholine [↓], glycerophosphoethanolamines [↓], multiple lipids [↓]	No other tests
								Het		NM_006623.4	c.518G>A	NP_006614.2:p.Gly173Glu	CA341849741	N/A	VUS		
971	F	13	Mild intellectual disability, chronic kidney disease with recent acute progression and scoliosis and feeding via a NG-tube	Yes	Methylmalonic aciduria due to methylmalonyl-CoA mutase deficiency	251000	*MUT*	Het	AR	NM_000255.4	c.1106G>A	NP_000246.2:p.Arg369His	CA312764	203846	P	Methylmalonate, propionylcarnitine, 2-methylcitrate	No other tests
								Het		NM_000255.4	c.1196_1197del	NP_000246.2:p.Val399GlufsTer24	CA567155985	578141	P		
987	M	13	Adrenal insufficiency, intellectual disability, short stature	Yes	Glycerol kinase deficiency; GKD	307030	*GK*	Hem	XLR	N/A	GRCh37/hg19 arr Xp21.3-p21.2(chrX:27958335-30700654)x0	N/A	N/A	N/A	P	Glycerol, multiple long chain and very long chain monoacylglycerols	No other tests
1028	M	1	Delayed speech, dysmorphic features, failure to thrive, hepatosplenomegaly and skin anomalies	Yes	Transaldolase deficiency; TALDOD	606003	*TALDO1*	Het	AR	NM_006755.2	c.512_514del	NP_006746.1:p.Ser171del	CA118881	7562	P	Ribitol, ribonate, erythronate, arabitol, erythritol, sedoheptulose	Polyols-U: sedoheptulose, arabitol, erythritol, ribitol
								Het		NM_006755.2	c.931G>T	NP_006746.1:p.Gly311Trp	CA378938636	625864	P		
1034	M	12	Congenital malformation syndrome associated with short stature, chronic bilateral blepharoconjunctivitis, eczema, cradle cap, and intellectual disability	Yes	Short stature, developmental delay, and congenital heart defects; SDDHD	617044	*TKT*	Hom	AR	NM_001064.4	c.769_770insCTACCTCCTTATCTTCTG	NP_001055.1:p.Trp257delinsSerThrSerLeuSerSerGly	CA358413	243092	P	Rbitol, ribonate, erythronate, erythritol, arabonate/xylonate, arabitol/xylitol, ribose	No other tests
1094	F	13	Hyperammonemia	No	Ornithine transcarbamylase deficiency, hyperammonemia due to	311250	*OTC*	Het	XLR	NM_000531.6	c.905A>G	NP_000522.3:p.His302Arg	CA224825	97352	P	Orotate, ornithine, N-carbamoylaspartate, uridine, uracil, alanine, aspartate, glutamine	PAA: citrulline [↓], glutamine, alanine and ornithine<br>UAA: alanine, ornithine, lysine, arginine<br>GAMT-P: -<br>OROT-U: -
1120	M	23	Progressive neuropathy	Yes	Mitochondrial DNA depletion syndrome 1 (MNGIE type); MTDPS1	603041	*TYMP*	N/A	AR	NM_001113755.2	c.215-1G>C	NP_001107227.1:p.=	CA212989	16662	P	Thymidine, thymine, 5,6-dihydrothymine [↓]	PDN-P: thymidine
1178	M	0.1	History of IUGR, mild elevated ammonia, weight loss, frequent spit-ups, increased emesis, and lactic acidosis	No	Methylmalonic aciduria due to methylmalonyl-CoA mutase deficiency	251000	*MUT*	Het	AR	NM_000255.4	c.1218del	NP_000246.2:p.Asn407ThrfsTer24	CA138796356	N/A	P	Methylmalonate, propionylcarnitine, propionylglycine, glycine, carnitine [↓]	Dry blood spot acylcarnitine profile: C3 acylcarnitine and C3/C2 ratio<br>UOA: methylmalonic acid, 3-hydroxypropionic acid, propionylglycine, methylcitric acid, lactic acid, pyruvic acid, benzoic acid, hippuric acid
								Het		NM_000255.4	c.1531C>T	NP_000246.2:p.Arg511Ter	CA3846855	556124	P		
1223	F	1.17	Neonatal onset propionic acidemia, developmental delay and hypotonia	Yes	Propionic acidemia	606054	*PCCA*	Hom	AR	NM_000282.4	c.425G>A	NP_000273.2:p.Gly142Asp	CA312819	203880	P	Propionylcarnitine, propionylglycine, methylcitrate, valine [↓], isoleucine [↓]	No other tests
1258	M	6	N.P	N.P	Beta-ureidopropionase deficiency; UPB1D	613161	*UPB1*	N.P	AR	N.P	N.P	N.P	N.P	N.P	N.P	3-ureidopropionate, 5,6-dihydrothymine, 5,6-dihydrouracil, 3-aminoisobutyrate [↓]	Metabolomics-U: 3-ureidopropionate, 5,6-dihydrothymine, 3-aminoisobutyrate [↓]
1274	F	15	History of two episodes of acute gastroenteritis in infancy	No	2-methylbutyryl-CoA dehydrogenase deficiency	610006	*ACADSB*	Hom	AR	NM_001609.4	c.1186A>G	NP_001600.1:p.Lys396Glu	CA5730953	377304	VUS	2-methylbutyrylcarnitine, 2-methylbutyrylglycine, 3-hydroxy-2-ethylpropionate, isobutyrylglycine, tiglylcarnitine	PCARN: -<br>PAA: alanine, ornithine, asparagine
1333	F	6	Global developmental delay, movement disorder	Yes	GABA-transaminase deficiency	613163	*ABAT*	Hom	AR	NM_020686.6	c.1394G>A	NP_065737.2:p.Gly465Asp	CA16607451	389161	VUS	2-pyrrolidinone, succinamic acid, succinimide	CSF neurotransmitters: -
1411	F	26	History of urocanic aciduria	No	Urocanase deficiency; UROCD	276880	*UROC1*	Het	AR	NM_144639.3	c.356C>G	NP_653240.1:p.Pro119Arg	CA2591587	N/A	P	Trans-urocanate, imidazole propionate, cis-urocanate	Newborn screening: urocanic acid<br>PAA: -
								Het		NM_144639.3	c.907G>C	NP_653240.1:p.Ala303Pro	CA2591263	N/A	P		
1441	M	5	History of chronic otitis media and reactive airways disease, speech delay	Yes	Acyl-CoA dehydrogenase, medium-chain, deficiency of; ACADMD	201450	*ACADM*	Hom	AR	NM_000016.6	c.-17C>G	NP_000007.1:p.=	CA912881	226052	VUS	N-palmitoylglycine, octanoylcarnitine, cis-4-decenoylcarnitine	Newborn screening: suspected medium chain acyl-CoA dehydrogenase (MCAD) deficiency, details were not provided
1450	M	28	Childhood onset of progressive spastic paraplegia	Yes	HSD10 mitochondrial disease; HSD10MD	300438	*HSD17B10*	Hem	XLD	N.P	N.P	N.P	N.P	N.P	N.P	Tiglylcarnitine, 2-hydroxy-3-methylvalerate, 3-beta-hydroxy-5-cholestanoate, multiple lipids, bile acids, tiglylglycine	No other tests
1461	M	4	Autism spectrum disorder	Yes	Autism, susceptibility to, X-linked 6; AUTSX6	300872	*TMLHE*	Hem	XLR	N/A	GRCh37/hg19 arr Xq28(chrX:154773423-154797292)x0	N/A	N/A	N/A	P	N6,N6,N6-trimethyllysine, carnitine [↓], deoxycarnitine [↓]	TML-P: trimethyllysine, gamma butyrobetaine [↓], carnitine [↓]<br>ACP: -<br>PCARN: -
1470	M	5	Global developmental delay, lower extremity hyperreflexia and spasticity, MRI brain with abnormal basal ganglia	Yes	Encephalopathy, ethylmalonic; EE	602473	*ETHE1*	Het	AR	NM_014297.5	c.263C>T	NP_055112.2:p.Ser88Leu	CA406179810	N/A	LP	Ethylmalonate, butyrylcarnitine, isobutyrylcarnitine, isovalerylcarnitine, glutarylcarnitine, 2-methylbutyrylcarnitine, methylsuccinate	Plasma lactate: -<br>Plasma ammonia: -<br>PAA: -<br>UOA: ethylmalonic acid<br>ACP: C4 acylcarnitine
								Het		NM_014297.5	c.407C>T	NP_055112.2:p.Thr136Ile	CA406175645	N/A	LP		
1683	F	0.25	Hyperargininemia, hyperammonemia, orotic aciduria, and possible global developmental delay	Yes	Argininemia	207800	*ARG1*	Het	AR	NM_000045.3	c.892G>C	NP_000036.2:p.Ala298Pro	CA3999402	419034	P	Pro-hydroxy-pro, arginine, dimethylarginine, trans-4-hydroxyproline, urea [↓]	Newborn screening: arginine<br>PAA: arginine<br>OROT-U: orotate<br>ARG-R: deficient
								Het		NM_000045.3	c.75A>G	NP_000036.2:p.Glu25=	CA3999170	355318	VUS		
1749	M	15	Intellectual disability and intractable generalized epilepsy	Yes	Adenylosuccinase deficiency; ADSLD	103050	*ADSL*	Het	AR	NM_000026.4	c.734G>A	NP_000017.1:p.Arg245Gln	CA10247801	625883	VUS	N6-succinyladenosine	No other tests
								Het		NM_000026.4	c.1393G>C	NP_000017.1:p.Val465Leu	CA411649151	625884	VUS		
1753	M	0	Liver dysfunction	No	Citrullinemia, type II, neonatal-onset	605814	*SLC25A13*	Hom	AR	NM_014251.3	c.852_855del	NP_055066.1:p.Met285ProfsTer2	CA253673	225472	P	Citrulline, methionine, argininosuccinate, bilirubin, galactitol, cholesterol; homocitrulline, arginine, homoarginine, galactonate, N-acetylphenylalanine, phenyllactate, 4-hydroxyphenylpyruvate	Newborn screening: citrulline<br>PAA: citrulline, threonine, arginine, methionine<br>OROT-U: -
1757	M	2	Anemia, ataxia, chronic tachycardia, abnormal eye movements, respiratory failure	Yes	Brown-Vialetto-Van Laere syndrome 2; BVVLS2	614707	*SLC52A2*	Het	AR	NM_001363118.2	c.1016T>C	NP_001350047.1:p.Leu339Pro	CA343794	39577	P	Riboflavin (supplemented), pyridoxine, pyridoxate (normalized in response to riboflavin supplementation), medium chain (C6, C8, C10:1) and very long chain (C24) acylcarnitines, 2-hydroxyglutarate, methylsuccinate, ethylmalonate, kynurenine, N-formylanthranilic acid, kynurenate [↓], picolinate [↓], methionine sulfone [↓]	ACP: C6 and C8 acylcarnitines<br>UOA: ethylmalonic acid<br>Metabolomics-CSF: octanoylcarnitine (C8), 5-oxoproline, tryptophan, isocitrate [↓], urea [↓], homoarginine [↓]<br>Metabolomics-U: riboflavin, pyridoxine, pyridoxate, 3-hydroxykynurenine, kynurenine, ornithine, N-delta acetylornithine, biopterin metabolites (including dihydrobiopterin, pterin, biopterin and neopterin), homoarginine [↓], homocitrulline [↓], urea [↓]
								Het		NM_001363118.2	c.405_407del	NP_001350047.1:p.Phe135del	CA848870182	N/A	P		
1797	F	0.75	Infantile spasms and global developmental delay	Yes	AICA-ribosiduria due to ATIC deficiency	608688	*ATIC*	Het	AR	NM_004044.7	c.1277A>G	NP_004035.2:p.Lys426Arg	CA119095	7810	P	N6-succinyladenosine	Metabolomics-CSF: N6-succinyladenosine, hypoxanthine [↓]
								Het		NM_004044.7	c.1113C>A	NP_004035.2:p.Tyr371Ter	CA350484015	N/A	P		
1800	F	1.25	Poor oral intake resulting in hypoglycemia with short fasting duration and G-tube feeding, ketosis, short stature and vomiting	No	Acyl-CoA dehydrogenase, medium-chain, deficiency of; ACADMD	201450	*ACADM*	Het	AR	NM_000016.6	c.799G>A	NP_000007.1:p.Gly267Arg	CA252824	3588	P	Octanoylcarnitine, hexanoylcarnitine	Metabolomics-U: C6, C8, C10 acylcarnitines
								Het		NM_000016.6	c.928G>A	NP_000007.1:p.Gly310Arg	CA274925	193539	P		
33	M	32	Seizures, mild intellectual disability, and a low protein diet	Yes	Argininemia	207800										N-acetylarginine, uridine, arginine, glutamine, 5,6-dihydrouracil, urea [↓]	GAMT-P: guanidinoacetate<br>PAA: arginine

© 2021 Liu N et al. *JAMA Network Open*.

Family[a] Number	Gender	Age (years)	Phenotype	Neuro[b]	Associated IEM	MIM#	Gene	Zygosity	Inheritance	Gene Accession ID, GRCh 37 (MANE Select)	Nucleotide Change	Amino Acid Change	Canonical Allele Identifier	Variation ID (ClinVar)	Pathogenicity (Clinical reports)	Metabolomic Findings	Other Biochemical Testing Findings
51	M	16	Chronic diarrhea, abdominal pain, poor weight gain	No	Propionic acidemia	606054										Propionylcarnitine, propionylglycine, 2-methylcitrate, glycine	UOA: methylcitric acid<br>PAA: glycine<br>ACP: C3 acylcarnitine
57	M	39	Global developmental delay, esotropia, retinal dystrophy, possible learning disability, eczema, chronic kidney disease, heart block with chronic a-fib, hypercholesterolemia, scoliosis, severe pectus excavatum, asthenic body habitus, apathy with long face, poor muscle strength, poor coordination, ataxia, diagnosis of cardiomyopathy at age 14 and transient ischemic attack at age 38	Yes	Cerebral creatine deficiency syndrome 2; CCDS2	612736										Guanidinoacetate, creatine [↓]	GAMT-P: guanidinoacetate
86	F	40	Hypertension, fatigue and history of seizures	Yes	Maple syrup urine disease; MSUD	248600										Leucine, N-acetylleucine, isoleucine, N-acetylisoleucine, valine, 2-hydroxy-3-methylvalerate, alpha-hydroxyisovalerate, alpha-hydroxyisocaproate, 4-methyl-2-oxopentanoate, 3-methyl-2-oxobutyrate, 3-methyl-2-oxovalerate, alpha-hydroxycaproate, beta-hydroxyisovalerate, isovalerate	PAA: leucine, isoleucine, valine, alloisoleucine<br>UOA: 2-hydroxyisovaleric acid, 2-hydroxy 3-methyl valeric acid
223	M	6	Unspecified delay in development, seizures, and speech delay	Yes	Propionic acidemia	606054										Propionylcarnitine, propionylglycine, 2-methylcitrate, glycine	PAA: glycine<br>ACP: C3 acylcarnitine<br>PCARN: free and total carnitines
347	M	13	Failure to thrive and short stature, developmental delay, single kidney	Yes	Cerebral creatine deficiency syndrome 2; CCDS2	612736										Guanidinoacetate, creatine [↓]	GAMT-P: guanidinoacetate; creatine [↓]
557	F	30	Previously diagnosed with hyperornithinemia-hyperammonemia-homocitrullinemia syndrome	N.P	Hyperornithinemia-Hyperammonemia-Homocitrullinuria syndrome; HHHS	238970										Ornithine, homocitrulline, N-delta-acetyl-ornithine	No other tests
672	F	13	Scleroderma and weight loss	No	Phenylketonuria; PKU	261600										Phenylalanine, phenylpyruvate, phenyllactate (PLA), 3-(4-hydroxyphenyl) lactate (HPLA), N-acetylphenylalanine, N-formylphenylalanine	No other tests
791	F	18	Intellectual disability, chronic constipation, abdominal pain, and vomiting	Yes	Tyrosinemia, type I; TYRSN1	276700										3-4-hydroxyphenyllactate (HPLA), 4-hydroxyphenylpyruvate, phenyllactate, tyrosine, N-acetyltyrosine	No other tests
792	M	15	Developmental delay, autistic features, feeding difficulties, lethargy, peripheral neuropathy, fatigue, ptosis, pseudo-obstruction, respiratory failure, organic aciduria, optic atrophy and increased signal basal ganglia	Yes	Methylmalonic aciduria due to methylmalonyl-CoA mutase deficiency	251000										Propionylcarnitine, 2-methylcitrate	PUR-U: -<br>PYR-U: -<br>PDN-P: -
916	M	18	Previously diagnosed with glutaric aciduria I deficiency, details were not provided	N.P	Glutaric acidemia I; GA1	231670										Glutaroylcarnitine, glutarate	No other tests
1035	F	10	Body odor, selective mutism, deafness and apparent attention deficit hyperactivity disorder	Yes	Isovaleric acidemia; IVA	243500										Isovalerylcarnitine, 2-methylbutyroylcarnitine, isovalerylglycine, isovalerate	No other tests
1091	F	12	Dystonia, bilateral hip dislocation and cognitive impairment	Yes	Glutaric acidemia I; GA1	231670										Glutaroylcarnitine, glutarate, multiple carnitine species [↓]	No other tests
1121	M	12	Gastroesophageal reflux disease, hearing problem, optic problem, mild intellectual disability, a bicuspid aortic valve, a bifid uvula, pes planus, hypotonia, developmental delay	Yes	Methylmalonic aciduria due to methylmalonyl-CoA mutase deficiency	251000										Methylmalonate, 2-methylcitrate, propionylcarnitine	No other tests
1191	M	26	Previously diagnosed with phenylketonuria (PKU) but non-responsive to therapy	N.P	Phenylketonuria; PKU	261600										Phenylalanine, phenyllactate, phenylpyruvate, N-acetylphenylalanine, N-formylphenylalanine, 3-(4-hydroxyphenyl)lactate (HPLA)	No other tests
1222	M	0.01	Status epilepticus, intubated, continuous EEG monitoring, and AED therapy	Yes	Carnitine deficiency, systemic primary; CDSP	212140										Carnitine and related carnitine conjugates [↓]	PAA: -<br>UOA: -<br>ACP: free carnitine and multiple acylcarnitines [↓]
1299	M	12	Microcephaly and history of bicuspid aortic valve, encopresis, high frequency hearing loss, intellectual disability and behavioral problems	Yes	Phenylketonuria; PKU	261600										Phenylalanine, phenylpyruvate, N-acetylphenylalanine, N-formylphenylalanine, phenyllactate	PAA: phenylalanine, citrulline, tyrosine [↓], leucine [↓], valine [↓]
1302	M	7	Feeding difficulties, balance problems, and high glutamine but normal ammonia	No	Lysinuric protein intolerance; LPI	222700										Arginine [↓], urea [↓], ornithine [↓], lysine [↓], dimethylarginine [↓]	Plasma ammonia: -<br>Metabolomics-U: homoarginine, N-acetylarginine, arginine, N-alpha-acetylornithine, urea [↓], homocitrulline [↓]
1489	F	63	Memory loss, history of depression, anxiety, diverticulitis, hypothyroidism, weight gain, cataract post-surgery	Yes	Galactosemia I; GALAC1	230400										Galactitol, galactonate	Metabolomics-U: galactitol
1519	F	39	Previously diagnosed with classic galactosemia, details not provided	N.P	Galactosemia I; GALAC1	230400										Galactitol, galactonate	Metabolomics-U: galactitol
1532	M	32	Vomiting, dizziness, cirrhosis, gastroparesis, obstructive sleep apnea, dilated cardiomyopathy, obesity, lower extremity edema	No	Multiple acyl-CoA dehydrogenase deficiency; MADD	231680										Glutarate, 2-hydroxyglutarate, glutarylcarnitine, a wide variety of various acylcarnitines	ACP (external): consistent with glutaric acidemia II, details not provided
1602	M	17	Previously diagnosed with MCAD with other concerns including developmental delay, episodes of pain	Yes	Acyl-CoA dehydrogenase, medium-chain, deficiency of; ACADMD	201450										Octanoylcarnitine, hexanoylcarnitine, cis-4-decenoylcarnitine, caproate, multiple medium chain fatty acids	ACP: C6 and C8 acylcarnitines<br>Metabolomics-U: heptanoyl glycine, phenylpropionylglycine, hexanoylglycine, octanoylcarnitine, hexanoylcarnitine
1603	F	5	Gait disturbance and slurred speech with evidence of bilateral basal ganglia abnormalities and lactic acidosis	Yes	Encephalopathy, ethylmalonic; EE	602473										Ethylmalonate, isobutyrylglycine, isobutyrylcarnitine, isovalerylcarnitine, 2-methylbutyrylcarnitine, butyrylcarnitine, 3-indoxyl sulfate [↓], glycolithocholate sulfate [↓]	Metabolomics-U: ethylmalonate, isobutyrylglycine, 2-methylbutyrylcarnitine
1648	F	1.42	Gross motor delay, and truncal and limb ataxia	Yes	Maple syrup urine disease; MSUD	248600										Leucine, valine, isoleucine, 4-methyl-2-oxopentanoate (ketoleucine), 3-methyl-2-oxovalerate	No other tests
**Subgroup B. 5 cases screened by both clinical metabolomics and traditional screening and both findings are consistent with diagnosis of an IEM**																	
522	M	9	Dehydration, altered mental status, hyperammonemia and migraines	Yes	Multiple acyl-CoA dehydrogenase deficiency	231680	*ETFDH*	Het<br>Het	AR	NM_004453.4<br>NM_004453.4	c.1832G>A<br>c.79C>T	NP_004444.2:p.Gly611Glu<br>NP_004444.2:p.Pro27Ser	CA3122732<br>CA233953	265124<br>167040	P<br>VUS	glutarate, glutaroylcarnitine, myristoylcarnitine, isovalerylglycine, ethylmalonate	UOA: 3-hydroxybutyric acid, acetoacetic acid, glutaric acid<br>ACP: numerous acylcarnitines, free carnitine [↓]<br>OROT-U: -
829	M	1	Hyperammonemia with seizures, lethargy, encephalopathy	Yes	Argininemia	207800	*ARG1*	Hom	AR	NM_000045.3	c.466-1G>C	NP_000036.2:p.=	CA16618244	419876	P	N-acetylarginine, arginine, 4-guanidinobutanoate, uridine, homoarginine, N-acetylcitrulline, homocitrulline, uracil	PAA: arginine<br>UOA: -<br>OROT-U: orotic acid<br>ACP: free carnitine [↓]<br>ARG-R: deficient
1006	F	1	Developmental delay and seizures	Yes	Citrullinemia, classic	215700	*ASS1*	Hom	AR	NM_054012.4	c.830A>G	NP_446464.1:p.Lys277Arg	CA375229529	N/A	LP	Citrulline, N-acetylcitrulline	PAA: citrulline<br>UOA: glutaric acid, 5-oxoproline<br>ACP: free carnitine [↓]<br>ETC-M: citrate synthase activity was reduced

© 2021 Liu N et al. *JAMA Network Open*.

Family[a] Number	Gender	Age (years)	Phenotype	Neuro[b]	Associated IEM	MIM#	Gene	Zygosity	Inheritance	Gene Accession ID, GRCh 37 (MANE Select)	Nucleotide Change	Amino Acid Change	Canonical Allele Identifier	Variation ID (ClinVar)	Pathogenicity (Clinical reports)	Metabolomic Findings	Other Biochemical Testing Findings
1246	F	1.08	Developmental delay, chronic diarrhea, easy petechial rash/bruising, failure to thrive	Yes	Encephalopathy, ethylmalonic; EE	602473	ETHE1	Hom	AR	NM_014297.4	Exon 4 del	Exon 4 del	N/A	2323	P	Ethylmalonate, butyrylcarnitine, isobutyrylcarnitine, isovalerylcarnitine, glutarylcarnitine, 2-methylbutyrylcarnitine, methylsuccinate, methylmalonate, phenol sulfate [↓]	ACP: C4 acylcarnitine<br>UOA: ethylmalonic acid, methylsuccinic acid, fumaric acid, citric acid<br>PAA: -<br>TPH-P: -<br>ACYG-U: isobutyrylglycine, 2-methybutyrylglycine
469	M	18	Intellectual disability, epilepsy, and gait problems	Yes	L-2-hydroxyglutaric aciduria; L2HGA	236792										2-hydroxyglutarate	PAA: -<br>ACP: -<br>UOA: 2-hydroxyglutaric acid
**Subgroup C. 6 cases screened by both clinical metabolomics and traditional screening in this study with metabolomic findings in support of the diagnosis of IEMs, while traditional screening was unrevealing.**																	
171	F	17	History of hypoglycemia, lactic acidemia, hyperammonemia, hypokalemia, hypophosphatemia, epilepsy, autism spectrum disorder, sensory integration disorder, and agenesis of corpus callosum	Yes	Neurodegeneration, infantile-onset, biotin-responsive; NERIB	618973	*SLC5A6*	Het	AR	NM_021095.4	c.1865_1866del	NP_066918.2:p.Gln622ArgfsTer51	CA1571320	N/A	VUS	Pantothenate (Vitamin B5) [↓], carnitine [↓], multiple carnitine derivatives [↓]	PAA: -<br>UOA: lactic acid, ketones, 3-OH isovaleric acid, glutaric acid, dicarboxylic acid<br>ACP: free carnitine [↓]<br>PMMA: -
								Het		NM_021095.4	c.814G>A	NP_066918.2:p.Gly272Arg	CA346174092	N/A	VUS		
584	M	3	Developmental delay, ocular motor apraxia, and seizures	Yes	Adenylosuccinase deficiency; ADSLD	103050	*ADSL*	Hom	AR	NM_000026.4	c.1277G>A	NP_000017.1:p.Arg426His	CA115565	2462	P	N6-succinyladenosine	PUR-U: succinyladenosine<br>PAA, ACP, GAMT-P, UCT-U, UOA, PYR-U: -
933	F	0.06	Intermittent spastic movements and fever likely secondary to meningoencephalitis infections	Yes	Epilepsy, pyridoxine-dependent; EPD	266100	*ALDH7A1*	Het	AR	NM_001182.5	c.834G>A	NP_001173.2:p.Val278=	CA3389640	18002	P	Pipecolate, 6-oxopiperidine-2-carboxylate	PAA: -<br>CSFAA: -<br>ACP: free carnitine [↓]
								Het		NM_001182.5	c.1279G>C	NP_001173.2:p.Glu427Gln	CA258070	17994	P		
576	M	1	Born prematurely with speech delay, global developmental delay, seizure disorder, spasticity, truncal hypotonia, dry skin and hearing loss	Yes	Autism, susceptibility to, X-linked 6; AUTSX6	300872										N6,N6,N6-trimethyllysine, deoxycarnitine (gamma butyrobetaine) [↓]	TML-P: trimethyllysine, gamma-butyrobetaine [↓]<br>ACP, PAA, UOA, UCT-U: -
704	F	5	Turner syndrome, cyclic vomiting syndrome, hyperemesis with high anion gap metabolic acidosis and hypoglycemia	No	Carnitine deficiency, systemic primary; CDSP	212140										Carnitine and related carnitine conjugates [↓]	PAA: -<br>UOA: 3-hydroxybutyric acid, acetoacetic acid<br>PCARN: free and total carnitine [↓]<br>ACP: free carnitine [↓]
1550	F	19	Encephalopathy and developmental regression over the last 4 years with new onset seizures, abnormal MRI findings, and multiple left renal cysts	Yes	Cerebral creatine deficiency syndrome 2; CCDS2	612736										Guanidinoacetate, creatine [↓]	GAMT-P: guanidinoacetate, creatine [↓]<br>PAA: alanine, citrulline, ornithine, cysteine<br>UOA: -<br>ACP: -<br>PMMA: -<br>UCT-U: -
**Group 2. 40 cases with metabolomic abnormalities suggestive for IEMs and further confirmed by available sequencing tests, biochemical targeted tests, and clinical phenotypes**																	
**Subgroup A. 31 cases screened by clinical metabolomics only and not screened by traditional screening approach in this study**																	
39	F	8	Delayed motor and speech, seizures, ataxia, abnormal movements, intellectual disability, and dystonia	Yes	Developmental and epileptic encephalopathy 25, with amelogenesis imperfecta; DEE25	615905	*SLC13A5*	Hom	AR	NM_177550.5	c.511del	NP_808218.1:p.Glu171SerfsTer16	CA16043136	372505	P	Citrate	UOA: citric acid
50	M	11	Frequent vomiting, poor tone, poor coordination and developmental delay currently feeding via G-tube	Yes	Mitochondrial DNA depletion syndrome 9 (encephalomyopathic type with methylmalonic aciduria); MTDPS9	245400	*SUCLG1*	Hom	AR	NM_003849.4	c.473A>G	NP_003840.2:p.Lys158Arg	CA1736305	N/A	Unclassified	Succinylcarnitine, propionylcarnitine, methylmalonate, hexanoylcarnitine, butyrylcarnitine, malate [↓]	ACP: C3 and C4OH acylcarnitines<br>UOA: methylmalonic acid, ethylmalonic acid, methylcitric acid, 3-hydoxyisovleric acid
60	F	22	Microcephaly, involuntary movements, hypertonia, profound cognitive deficits, autistic traits, sleep disturbances and seizures	Yes	Spastic paraplegia 9B, autosomal recessive; SPG9B	616586	*ALDH18A1*	Hom	AR	NM_002860.4	c.2294G>A	NP_002851.2:p.Arg765Gln	CA210009	216888	LP	Proline [↓], ornithine [↓]	No other tests
70	F	14	Delayed motor milestones, delayed speech, developmental regression, intellectual disability, and abnormal movements	Yes	3-hydroxyisobutyryl-CoA hydrolase deficiency; HIBCHD	250620	*HIBCH*	Hom	AR	NM_014362.4	c.196C > T	NP_055177.2:p.Arg66Trp	CA199654	190268	VUS	3-hydroxyisobutyrate [↓]	No other tests
177	F	8	Hyperammonemia and altered mental status with associated severe head pain, difficulty moving left side, twitching of mouth and arm. Brain MRI and EEG were abnormal	Yes	Ornithine transcarbamylase deficiency, hyperammonemia due to	311250	*OTC*	Het	XLR	NM_000531.6	c.731T>C	NP_000522.3:p.Leu244Pro	CA412722291	N/A	VUS	Orotate	PAA: glutamine<br>OROT-U: orotate
207	M	8	Developmental delay, short stature, dysmorphic features, narrow face, high arched palate, syndactyly, and asthma	Yes	Glutaric aciduria III; GA3	231690	*SUGCT*	Het	AR	NM_001193313.2	c.152+1G>A	NP_001180242.2:p.=	CA4229337	N/A	P	Glutarate, arachidate (20:0), octadecadienedioate (C18:2-DC), hexadecanedioate, octadecenedioate (C18:1-DC), ximenoylcarnitine (C26:1)	UOA: glutaric acid, succinic acid<br>PAA: -<br>ACP: -
								Het		NM_001193313.2	c.676A>G	NP_001180242.2:p.Thr226Ala	CA367312696	N/A	VUS		
252	M	12	Developmental regression, joint contractures, scoliosis, nystagmus	Yes	Peroxisome biogenesis disorder 8A (Zellweger); PBD8A	614876	*PEX16*	Hom	AR	NM_004813.2	c.859C>T	NP_004804.1:p.Arg287Cys	CA5959797	561075	VUS	Pipecolate, phytanate, long chain fatty acids,1-lignoceryl-GPC, 7-HOCA, hexadecanedioate, octadecanedioate, eicosanodioate, docosadioate, phytanate, multiple sphingomyelins [↓], plasmalogens [↓], phosphatidylcholines [↓], phosphatidylethanolamines [↓]	Very long chain fatty acids: C26:0 and C:26:0/C22:0
315	M	0.06	Hypotonia and lethargy	No	Methylmalonic aciduria and homocystinuria, cblC type; MAHCC	277400	*MMACHC*	Het	AR	NM_015506.3	c.271dup	NP_056321.2:p.Arg91LysfsTer14	CA090937	1421	P	Methylmalonic acid, 3-hydroxy-3-methylglutaric acid, propionylcarnitine, methionine [↓]	Newborn screening: C3 acylcarnitine, methylmalonic acid, homocysteine, methionine [↓]<br>ACP: C3 acylcarnitine<br>PMMA: methylmalonic acid<br>PAA: methionine [↓]<br>UOA: methylmalonic acid<br>TPH-P: homocysteine
								Het		NM_015506.3	c.331C>T	NP_056321.2:p.Arg111Ter	CA251789	1424	P		
								Het		NM_015506.3	c.181C>T	NP_056321.2:p.Arg61Trp	CA312726	203824	VUS		
361	F	6	Motor milestones, delayed speech and language, bilateral sensorineural hearing loss, retinitis pigmentosa and optic nerve hypoplasia	Yes	Peroxisome biogenesis disorder 1A (Zellweger); PBD1A	214100	*PEX1*	Hom	AR	NM_000466.3	c.2528G>A	NP_000457.1:p.Gly843Asp	CA220977	7516	P	Pipecolate, docosadioate, multiple sphingomyelins [↓]	No other tests
393	M	3	Recurrent episode of cranial nerve palsy and ataxia	Yes	Pyruvate dehydrogenase, alpha-1; PDHA1	300502	*PDHA1*	Hem	XLD	NM_000284.4	c.832G>A	NP_000275.1:p.Gly278Arg	CA16608805	384555	P	Lactate, pyruvate, alanine	PAA: alanine<br>UOA: -
430	M	17	Motor and speech delay, intellectual disability and spasticity	Yes	Encephalopathy, ethylmalonic; EE	602473	*ETHE1*	Hom	AR	NM_014297.5	c.79C>A	NP_055112.2:p.Gln27Lys	CA9487970	617791	P	Ethylmalonate, several C4 and C5 acylcarnitines, methylsuccinate, methylmalonate	No other tests
440	F	1	Significant hypotonia, global developmental delay, and seizures. He has dysmorphic features with long forehead, low set and malformed ears and small mouth and significant hepatosplenomegaly	Yes	Peroxisome biogenesis disorder 4A (Zellweger); PBD4A	614862	*PEX6*	Hom	AR	NM_000287.4	c.611C>G	NP_000278.3:p.Ser204Ter	CA3811598	N/A	P	Pipecolate, 1-lignoceryl-GPC, 7-HOCA, hexadecanedioate, octadecanedioate, eicosanodioate, docosadioate, phytanate, multiple sphingomyelins [↓], plasmalogens [↓], phosphatidylcholines [↓], phosphatidylethanolamines [↓]	UOA: 4-hydroxyphenyllactic acid
514	M	0.17	Seizures, hypotonia, and global developmental delay	Yes	Glycine encephalopathy; GCE	605899	*GLDC*	Het	AR	NM_000170.3	c.1009C>T	NP_000161.2:p.Arg337Ter	CA263279	56036	P	Glycine	CSFAA: glycine and CSF/plasma ratio<br>PAA: glycine
								Het		NM_000170.3	c.2293C>T	NP_000161.2:p.Pro765Ser	CA263353	56070	LP		
721	M	1	Succinic semialdehyde dehydrogenase (SSADH) deficiency: baseline axial hypotonia with intermittent	Yes		271980	*ALDH5A1*	Het	AR	NM_001080.3	c.1015-2A>C	NP_001071.1:p.=	CA3656837	N/A	P	2-pyrrolidinone, 4-guanidinobutanoate	PAA: glycine

© 2021 Liu N et al. *JAMA Network Open*.

Family[a] Number	Gender	Age (years)	Phenotype	Neuro[b]	Associated IEM	MIM#	Gene	Zygosity	Inheritance	Gene Accession ID, GRCh 37 (MANE Select)	Nucleotide Change	Amino Acid Change	Canonical Allele Identifier	Variation ID (ClinVar)	Pathogenicity (Clinical reports)	Metabolomic Findings	Other Biochemical Testing Findings
			episodic worsening, gross motor developmental delay and gastroesophageal reflux disorder		Succinic semialdehyde dehydrogenase deficiency; SSADHD			Het		NM_001080.3	c.1597G>A	NP_001071.1:p.Gly533Arg	CA3656974	426524	P		UOA: 4-OH butyric acid, 2,4-dihydroxybutyric acid, 3,4-dihydroxybutyric acid
833	M	1	Suspected acyl-CoA dehydrogenase, short-chain, deficiency, elevated C4 on NBS	N.P	Acyl-CoA dehydrogenase, short-chain, deficiency of; ACADSD	201470	*ACADS*	Hom	AR	NM_000017.4	c.625G>A	NP_000008.1:p.Gly209Ser	CA145599	3831	P	Ethylmalonate	Newborn screening: C4 acylcarnitine
847	F	16	Chronic transaminase elevations, cognitive delay and myopia	Yes	Alpha-methylacyl-CoA racemase deficiency; AMACRD	614307	*AMACR*	Hom	AR	NM_014324.5	c.877T>C	NP_055139.4:p.Cys293Arg	CA359399111	N/A	P	Phytanate, 7-alpha-hydroxy-3-oxo-4-cholestenoate (7-HOCA)	No other tests
854	F	7	Intellectual disability and speech delay	Yes	HSD10 mitochondrial disease; HSD10MD	300438	*HSD17B10*	Het	XLD	NM_004493.3	c.753C>G	NP_004484.1:p.Ile251Met	CA413150051	N/A	LP	Tiglylcarnitine, tiglylglycine	ACYG-U: tiglylglycine<br>PAA: -<br>UOA: -
902	M	31	Vomiting, stomach problems, nausea, gastroesophageal reflux, possible delayed gastric emptying, chronically low body weight and increased urinary lactate	Yes	Fructose intolerance, hereditary; HFI	229600	*ALDOB*	Het	AR	NM_000035.4	c.448G>C	NP_000026.2:p.Ala150Pro	CA339810	464	P	Fructose	No other tests
								Het		NM_000035.4	c.488C>T	NP_000026.2:p.Ala163Val	CA5161586	389159	VUS		
958	M	0.08	Infantile seizures, mild hypotonia, episodic apnea, mild dysmorphic features	Yes	D-bifunctional protein deficiency	261515	*HSD17B4*	Het	AR	NM_000414.4	c.936_937del	NP_000405.1:p.Thr313Ter	CA3382041	504023	P	1-lignoceroyl-GPC (24:0), docosadienoate, sphingomyelins [↓], phosphatidylcholines [↓]	AASA-CSF: pipecolic acid<br>TPH-P: methionine
								Het		NM_000414.4	c.1210-11C>G	NP_000405.1:p.=	CA3382130	559071	VUS		
1063	M	1	Phosphoglycerate dehydrogenase deficiency with a history of anemia, growth failure, developmental delay, IUGR, microcephaly, increased toned and ichthyosis	Yes	Phosphoglycerate dehydrogenase deficiency; PHGDHD	601815	*PHGDH*	Hom	AR	NM_006623.4	c.1286G>T	NP_006614.2:p.Gly429Val	CA341853632	N/A	LP	Serine [↓], glycine [↓], sphingomyelins [↓], glycerophosphocholines [↓], glycerophosphoethanolamines [↓]	PAA: serine [↓], glycine [↓]<br>CSFAA: serine [↓], glycine [↓]
1253	F	0.42	Severe feeding difficulty, failure to thrive, pan-craniosynostosis and near-constant implacable crying, profound developmental delay. Abnormal EEG with diffuse low voltage and a paucity of cerebral activity	Yes	Phosphoserine aminotransferase deficiency; PSATD	610992	*PSAT1*	Het	AR	NM_058179.4	c.432del	NP_478059.1:p.Asp145Metfs Ter49	CA658657871	450376	P	Serine [↓], glycine [↓], glycerophosphocholines [↓], glycerophosphoethanolamines [↓], multiple lipids [↓]	PAA: serine [↓], glycine [↓]<br>CSFAA: serine [↓], glycine [↓]
								Het		NM_058179.4	c.44C>T	NP_478059.1:p.Ala15Val	CA373871234	450380	P		
1449	M	14	Cataracts, very subtle cutis laxa, plus small size and developmental delay, arginine supplementation	Yes	Spastic paraplegia 9B, autosomal recessive; SPG9B/Cutis laxa type IIIA	616586/ 219150	*ALDH18A1*	Hom	AR	N.P	N.P	N.P	N.P	N.P	N.P	Citrulline [↓], multiple alterations of bile acids, lipids, evidence of hepatic dysfunction	No other tests
1469	F	1	Failure to thrive, gross motor developmental delays, hypotonia, respiratory distress, and areflexia	Yes	Multiple mitochondrial dysfunctions syndrome 1; MMDS1	605711	*NFU1*	Het	AR	NM_001002755.4	c.622G>T	NP_001002755.1:p.Gly208Cys	CA129406	30700	P	Glycine, multiple plasmalogens [↓], phosphatidylcholines [↓], and sphingomyelins [↓]	UOA: -<br>PAA: glycine, mild<br>Metabolomics-CSF: glycine and lactate
								Het		NM_001002755.4	c.544C>T	NP_001002755.1:p.Arg182Trp	CA347123810	488564	VUS		
1483	M	2	Ventriculomegaly secondary to aqueductal narrowing, global developmental delay, abnormal tone, cerebral visual impairment, bilateral hip chondrodysplasia, schistocytosis, anemia, and thrombocytopenia	Yes	Spondyloepimetaphyseal dysplasia, Genevieve type; SEMDG	610442	*NANS*	Hom	AR	NM_018946.3	c.679G>A	NP_061819.2:p.Ala227Thr	CA374201023	N/A	VUS	N-acetylglucosamine/N-acetylgalactosamine, N-acetylneuraminate [↓]	No other tests
1486	M	20	Hearing loss, retinopathy, progressive peripheral neuropathy, cerebellar ataxia/atrophy, and mild cognitive decline	Yes	D-bifunctional protein deficiency	261515	*HSD17B4*	Het	AR	NM_000414.4	c.1547T>C	NP_000405.1:p.Ile516Thr	CA163183	137617	LP	Pristanate, phytanate (high normal)	No other tests
								Het		NM_000414.4	c.101C>T	NP_000405.1:p.Ala34Val	CA163180	137616	VUS		
1556	M	4	IUGR, hypertrophic cardiomyopathy, failure to thrive, developmental delay, dysmorphic features, and mild hypotonia	Yes	Mitochondrial complex V (ATP synthase) deficiency, nuclear type 2; MC5DN2	614052	*TMEM70*	Hom	AR	NM_017866.6	c.563T>C	NP_060336.3:p.Leu188Pro	CA371490258	N/A	VUS	3-methylglutaconate, 3-methylglutarylcarnitine, alanine, lactate	No other tests
1733	M	6	Autism, developmental delay, hypotonia, apraxia, dysmorphic features, and diarrhea	Yes	Isopentenyl-diphosphate delta isomerase 1; IDI1	604055	*IDI1*	Het	AR	NM_004508.2	c.229A>T	NP_004499.2:p.Met77Leu	CA5383736	N/A	VUS	Deoxycholate, 1-(1-enyl-stearoyl)-2-docosahexaenoyl-GPE (P-18:0/22:6), octadecadienedioate (C18:2-DC), dodecadienoate (12:2), 1-(1-enyl-palmitoyl)-2-linoleoyl-GPE (P-16:0/18:2), 1-(1-enyl-stearoyl)-2-linoleoyl-GPE (P-18:0/18:2), 1,2-dilinoleoyl-GPC (18:2/18:2), multiple sphingomyelins [↓]	No other tests
								Het		NM_004508.2	c.593_596del	NP_004499.2:p.Asp198Valfs Ter14	N/A	N/A	VUS		
62	F	18	Reported history of methylmalonic aciduria from NBS	No	Methylmalonic aciduria due to methylmalonyl-CoA mutase deficiency	251000										Methylmalonate	Newborn screening: consistent with methylmalonic aciduria, details not provided<br>PCARN: free and total carnitines<br>ETC-S: -<br>Urine fatty acid beta oxidation profile: ethylmalonic acid<br>ACP: C4 acylcarnitine
137	M	8	Previously diagnosed with dihydrolipoamide dehydrogenase deficiency with developmental delays, intellectual disability, abnormal movements, dietary avoidances, dysmorphic features, eye problems, failure to thrive, hypotonia, joint contractures, and organomegaly	Yes	Dihydrolipoamide dehydrogenase deficiency; DLDD	246900										Lactate, pyruvate, 3-methyl-2-oxobutyrate, 3-methyl-2-oxovalerate, 4-methyl-2-oxopentanoate, 2-hydroxyglutarate	No other tests
184	F	22	Previously diagnosed with carbamoyl-phosphate synthase 1 (CPS1) deficiency with reactive airways disease, cognitive impairment and seizures	Yes	Carbamoyl phosphate synthetase I deficiency, hyperammonemia due to	237300										Pyroglutamine, citrulline [↓]	Metabolomics-U: argininosuccinate, homocitrulline [↓], citrate [↓], succinate [↓]
1156	M	24	Seizure disorder, poor weight gain	Yes	Multiple acyl-CoA dehydrogenase deficiency; MADD	231680										Alpha-hydroxyisovalerate, 2-hydroxy-3-methylvalerate, 2-methylbutyrylglycine	Carnitine and acylcarnitine are suggestive of GAII, details were not provided
**Subgroup B. 2 cases screened by both clinical metabolomics and traditional screening approach in this study, consistent with IEM diagnosis.**																	
923	M	0.83	Short stature, failure to thrive (G-tube), acute kidney injury, megaloblastic anemia, thrombocytopenia, and lethargy	Yes	Homocystinuria-megaloblastic anemia, cblG complementation type; HMAG	250940	*MTR*	Het	AR	NM_000254.2	c.2405+1G>A	NP_000245.2:p.=	CA345379301	N/A	P	S-adenosylhomocysteine, methionine [↓]	ACP: C4 acylcarnitine<br>PAA: methionine [↓]<br>UOA: -
								Het		NM_000254.2	c.2473+3A>G	NP_000245.2:p.=	CA923726079	N/A	LP		
1072	M	4	History of intrauterine cardia rhabdomyoma and possible psychomotor regression. Brain MRI revealed bilateral basal ganglia lesions including caudate nuclei	Yes	HSD10 mitochondrial disease; HSD10MD	300438	*HSD17B10*	Hem	XLD	NM_004493.3	c.734T>C	NP_004484.1:p.Phe245Ser	CA413150197	N/A	VUS	Tiglylcarnitine, beta-hydroxyisovalerate, 3-hydroxyisobutyrate, isoleucine, 3-hydroxy-2-ethylpropionate, 2-methylbutyroylcarnitine, tiglylglycine	PAA: -<br>ACP: C5:1 acylcarnitine<br>UOA: lactic acid, 2-methyl-3-OH butyric acid, tiglylglycine
**Subgroup C. 7 cases screened by both clinical metabolomics and traditional screening approach in this study where metabolomic findings supported the diagnosis of IEMs but traditional screening was unrevealing**																	
41	M	9	Developmental delay, static encephalopathy, Chiari malformation, cerebellar ataxia, epilepsy and severe speech delay	Yes	Developmental and epileptic encephalopathy 25, with amelogenesis imperfecta; DEE25	615905	*SLC13A5*	Het	AR	NM_177550.5	c.997C>T	NP_808218.1:p.Arg333Ter	CA8331558	280534	P	Citrate	PAA: -<br>ACP: -<br>UOA: -<br>TPH-P: -<br>GAMT-P: -<br>UCT-U: -<br>PUR-U: -
								Het		NM_177550.5	c.680C>T	NP_808218.1:p.Thr227Met	CA210667	140753	P		
52	M	1	Delayed motor milestones, neonatal onset seizure disorder, ataxia, hypotonia, and abnormal movements	Yes	Developmental and epileptic encephalopathy 25, with amelogenesis imperfecta; DEE25	615905	*SLC13A5*	Het	AR	NM_177550.5	c.655G>A	NP_808218.1:p.Gly219Arg	CA210665	140752	P	Citrate	UOA: benzoic acid<br>PAA: -<br>ACP: -
								Het		NM_177550.5	c.1475T>C	NP_808218.1:p.Leu492Pro	CA16044235	375390	LP		
156	F	7	Progressive weakness, respiratory abnormalities, global developmental delay, aphasia, history of microcephaly and seizure, reported congenital cytomegalovirus infection (CMV)	Yes	Homocystinuria due to MTHFR deficiency	236250	*MTHFR*	Het	AR	NM_005957.5	c.1603C>T	NP_005948.3:p.Arg535Trp	CA595286	N/A	LP	Methionine sulfoxide [↓], betaine [↓], methionine [↓], methionine sulfone [↓]	ACP: free carnitine [↓]<br>TPH: homocysteine<br>PAA: -<br>UOA: -<br>PCARN: -
								Het		NM_005957.5	c.1529A>C	NP_005948.3:p.Lys510Thr	CA595336	N/A	VUS		

© 2021 Liu N et al. *JAMA Network Open*.

Family[a] Number	Gender	Age (years)	Phenotype	Neuro[b]	Associated IEM	MIM#	Gene	Zygosity	Inheritance	Gene Accession ID, GRCh 37 (MANE Select)	Nucleotide Change	Amino Acid Change	Canonical Allele Identifier	Variation ID (ClinVar)	Pathogenicity (Clinical reports)	Metabolomic Findings	Other Biochemical Testing Findings
358	M	1	Global developmental delay, hypotonia, macrocephaly and leukodystrophy	Yes	Lesch-Nyhan syndrome; LNS	300322	*HPRT1*	Hem	XLR	NM_000194.3	c.610C>G	NP_000185.1:p.His204Asp	CA254999	10047	LP	Inosine, uracil	PAA: arginine [↓]<br>ACP: C10, C10:1 acylcarnitines<br>UOA: 3-Hydroxybutyric acid, acetoacetic acid<br>ACP: C10:1, C10 acylcarnitines;<br>U-PUR: hypoxanthine
731	M	0.3	Lactic acidosis and seizures	Yes	Lipoyltransferase 1 deficiency; LIPT1D	616299	*LIPT1*	Het	AR	NM_145199.3	c.212C>T	NP_660200.1:p.Ser71Phe	CA199524	189835	LP	Beta-alanine, 4-guanidinobutanoate, methionine, isoleucine, leucine, 2-hydroxyadipate, tryptophan, lysine, phenylalanine, isovalerylcarnitine (C5), tyrosine, valine, 1-stearoyl-2-arachidonoyl-GPI (18:0/20:4), 3-hydroxy-3-methylglutarate, 2-aminoadipate, isovalerylglycine, arginine, serine oxalate (ethanedioate) [↓], malonate [↓]	ACP: free carnitine [↓]<br>UOA: lactic acid, succinic acid, fumaric acid, 2-hydroxyglutaric acid<br>PAA: alanine, proline, glutamate, arginine [↓]<br>PUR-U: hypoxanthine
								Het		NM_145199.3	c.539T>C	NP_660200.1:p.Leu180Ser	CA347853416	N/A	VUS		
1261	M	0.42	History of back arching and MRI concerning for Leigh syndrome	Yes	Mitochondrial short-chain enoyl-CoA hydratase 1 deficiency; ECHS1D	616277	*ECHS1*	Het	AR	NM_004092.4	c.538A>G	NP_004083.3:p.Thr180Ala	CA5765729	691284	P	Beta-hydroxyisovalerate, 1-lignoceroyl-GPC (24:0), 3-hydroxy-3-methylglutarate, and laurate (12:0)	ACP: -<br>PAA: -<br>UOA: -
								Het		NM_004092.4	c.444G>T	NP_004083.3:p.Met148Ile	CA216817873	N/A	VUS		
1347	M	0.58	Episodes of postprandial hypoglycemia and hepatomegaly	No	Glycogen storage disease IA; GSD1A	232200	*G6PC*	Het	AR	NM_000151.4	c.247C>T	NP_000142.2:p.Arg83Cys	CA256177	11998	P	Lactate, urate, glucose [↓], palmitoyl-linoleoyl-glycerol	ACP: C10:1 and C10 acylcarnitines<br>UOA, BIOT, PAA: -
								Het		NM_000151.4	c.379_380dup	NP_000142.2:p.Tyr128ThrfsTer3	CA256176	11997	P		
**Group 3. 4 cases with metabolomic abnormalities non-specific for IEMs and further confirmed by available sequencing tests, biochemical targeted tests, and/or clinical phenotypes**																	
34	F	19	Pre-syncopal episodes, occasional vomiting, and diarrhea	Yes	Mitochondrial complex V (ATP synthase) deficiency, nuclear type 2; MC5DN2	614052	*TMEM70*	Het	AR	NM_017866.6	c.317-2A>G	NP_060336.3:p.=	CA114347	540	P	3-methylglutarylcarnitine	ETC-S: deficiency of respiratory chain complex II<br>ACP: multiple acylcarnitines (medium and long chain)
								Het		NM_017866.6	c.494G>A	NP_060336.3:p.Gly165Asp	CA233205	156345	Unclassified		
341	M	0.25	Failure to thrive, Dandy-Walker malformation, cholestatic liver disease, undescended testes and failed newborn hearing screen	Yes	Adrenoleukodystrophy; ALD	300100	*ABCD1*	Hem	XLR	N/A	GRCh37/hg19 arr Xq28(chrX:152980470-153032459)x0	N/A	N/A	N/A	P	Numerous significant abnormalities in bile acids, fatty acids, and lipid metabolites	Bile acids: conjugated glycochenodeoxycholic acid
595	F	0.08	Metabolic acidosis, tachypnea, hyperglycemia, elevated lactate, hyperammonemia	No	Liver failure, infantile, transient; LFIT	613070	*TRMU*	Het	AR	NM_018006.5	c.117G>A	NP_060476.2:p.Trp39Ter	CA411939993	N/A	P	3-(4-hydroxyphenyl) lactate, phenyllactate, 4-hydroxyphenylpyruvate, N-acetylphenylalanine, fumarate, alanine, lactate, pyruvate, glycochenodeoxycholate, glycohycholate, taurocholate, bilirubin	PAA: glutamine, proline, citrulline, tyrosine, lysine, alanine<br>ACP: multiple short chain acylcarnitines
								Het		NM_018006.5	c.680G>C	NP_060476.2:p.Arg227Thr	CA10292164	N/A	VUS		
1390	M	0.1	Hyperbilirubinemia and coagulopathy likely due to liver failure	No	Congenital alloimmune liver disease, neonatal hemochromatosis	231100										Bilirubin, numerous lipids, bile acids, urea cycle metabolites, histidine, tryptophan, tyrosine, methionine, phenylalanine metabolites	Metabolomics-U: reflective of plasma metabolomics

[a]Family numbers are listed in order of the receipt date of initial plasma metabolomic sample. For those patients referred to our laboratory for repeat clinical metabolomic testing, the listed information including age, clinical presentation and metabolomic results are based on the clinical report of the initial sample.
[b]Neurological phenotype represents the symptoms that are caused by the dysfunction of nervous system which may include central nervous system and peripheral nervous system. Examples of symptoms include developmental delay, speech delay, autism spectrum disorder, intellectual disability, paralysis, seizures, spasticity, hypotonia, and poor coordination.
[c]Analytes listed represent elevations, unless otherwise indicated. Reductions, [↓]; (-), normal or no significant abnormalities in the testing result.

Abbreviations: N.P: Testing was performed in external labs, details were not provided on the requisition form; N/A: information is not available; CSF: cerebrospinal fluid; AASA-CSF: pyridoxine-dependent seizure panel in CSF (pipecolic acid, piperideine-6-carboxylate); ACP: plasma acylcarnitine profile; ACYG-U: urine acyl glycines panel; ARG-R: arginine enzyme assay (red blood cells); BIOT: serum biotinidase assay; CSFAA: CSF amino acids; GAMT-P: guanidinoacetate/creatine in plasma; OROT-U: urine orotic acid; PAA: plasma amino acids; PCARN: plasma carnitine; PDN-P: plasma thymidine; PUR-U: urine purine panel; TML-P: carnitine biosynthesis panel in plasma; TPH-P: total homocysteine in plasma; UCT-U: guanidinoacetate/creatine in urine; UOA: urine organic acids. Neuro, neurological phenotype

© 2021 Liu N et al. *JAMA Network Open*.

eTable 4. Categorized Metabolic Conditions Screened by Clinical Plasma Metabolomics and Covered by Traditional Screening and/or the RUSP

IEM #	Disease Category	IEM	OMIM	[a]IEMbase Nosology	Approach (No. of IEM)			
					Screened by Metabolomics (70 in total)			
					Metabolomics Only (28 IEM)		Metabolomics & Traditional (42 IEM)	
					Metabolomics Only – NOT Covered by RUSP (26)	Metabolomics Only - Covered by RUSP (2)	Metabolomics & Traditional – NOT Covered by RUSP (23)	Metabolomics & Traditional - Covered by RUSP (19)
1	Congenital disorders of glycosylation	Spondyloepimetaphyseal dysplasia, Genevieve type; SEMDG	610442	I.125.03	Yes			
2	Disorders of carbohydrates	Fructose intolerance, hereditary; HFI	229600	C.48.02	Yes			
3		Galactosemia I; GALAC1	230400	C.47.01		Yes		
4		Glycogen storage disease IA; GSD1A	232200	C.52.01	Yes			
5		Short stature, developmental delay, and congenital heart defects; SDDHD	617044	C.49.04	Yes			
6		Transaldolase deficiency; TALDOD	606003	C.49.03	Yes			
7	Disorders of lipids	Acyl-CoA dehydrogenase, medium-chain, deficiency of; ACADMD	201450	E.83.02				Yes
8		Acyl-CoA dehydrogenase, short-chain, deficiency of; ACADSD	201470	E.83.01				Yes
		IEM	OMIM	[a]IEMbase	Approach (No. of IEM)			

© 2021 Liu N et al. *JAMA Network Open*.

IEM #	Disease Category			Nosology	Screened by Metabolomics (70 in total)			
					Metabolomics Only (28 IEM)		Metabolomics and Traditional (42 IEM)	
					Metabolomics Only – NOT Covered by RUSP (26)	Metabolomics Only - Covered by RUSP (2)	Metabolomics & Traditional – NOT Covered by RUSP (23)	Metabolomics & Traditional - Covered by RUSP (19)
9	Disorders of lipids	Alpha-methylacyl-CoA racemase deficiency; AMACRD	614307	E.97.05	Yes			
10		Autism, susceptibility to, X-linked 6; AUTSX6	300872	E.82.05			Yes	
11		Carnitine deficiency, systemic primary; CDSP	212140	E.82.01				Yes
12		Glycerol kinase deficiency; GKD	307030	E.87.01			Yes	
13		Isopentenyl-diphosphate delta isomerase 1; IDI1	604055	N/A	Yes			
14		Smith-Lemli-Opitz syndrome; SLOS	270400	E.95.14	Yes			
15	Disorders of nitrogen-containing compounds	2-methylbutyryl-CoA dehydrogenase deficiency	610006	A.14.09				Yes
16		3-hydroxyisobutyryl-CoA hydrolase deficiency; HIBCHD	250620	A.14.14				Yes
17		Adenylosuccinase deficiency; ADSLD	103050	A.2.03	Yes			
18		AICA-ribosiduria due to ATIC deficiency	608688	A.2.21	Yes			
19		Argininemia	207800	A.7.06				Yes
20		Aromatic L-amino acid decarboxylase deficiency, AADCD	608643	A.10.02	Yes			
IEM #	Disease Types	IEM	OMIM	[a]IEMbase#	Approach (No. of IEM)			
					Screened by Metabolomics (70 in total)			

© 2021 Liu N et al. *JAMA Network Open*.

					Metabolomics Only (28 IEM)		Metabolomics and Traditional (42 IEM)	
					**Metabolomics Only – NOT Covered by RUSP (26)**	**Metabolomics Only - Covered by RUSP (2)**	**Metabolomics & Traditional – NOT Covered by RUSP (23)**	**Metabolomics & Traditional - Covered by RUSP (19)**
21	Disorders of nitrogen-containing compounds	Beta-ureidopropionase deficiency; UPB1D	613161	A.17.03	Yes			
22		Carbamoyl phosphate synthetase I deficiency, hyperammonemia due to	237300	A.7.02			Yes	
23		Cerebral creatine deficiency syndrome 2; CCDS2	612736	A.4.03	Yes			
24		Citrullinemia, classic	215700	A.7.04				Yes
25		Citrullinemia, type II, neonatal onset	605814	A.7.08				Yes
26		Dihydrolipoamide dehydrogenase deficiency; DLDD	246900	A.14.05			Yes	
27		Encephalopathy, ethylmalonic; EE	602473	A.13.10			Yes	
28		Epilepsy, pyridoxine-dependent; EPD	266100	A.15.02	Yes			
29		GABA-transaminase deficiency	613163	A.17.05	Yes			
30		Glutaric acidemia I; GA1	231670	A.15.05				Yes
31		Glutaric aciduria III; GA3	231690	A.15.06			Yes	
32		Glycine encephalopathy; GCE	605899	A.24.01			Yes	
**IEM #**	**Disease Types**	**IEM**	**OMIM**	**[a]IEMbase Nosology**	**Approach (No. of IEM)**			
					**Screened by Metabolomics (70 in total)**			

© 2021 Liu N et al. *JAMA Network Open*.

					Metabolomics Only (28 IEM)		Metabolomics and Traditional (42 IEM)	
					Metabolomics Only – NOT Covered by RUSP (26)	Metabolomics Only - Covered by RUSP (2)	Metabolomics & Traditional – NOT Covered by RUSP (23)	Metabolomics & Traditional - Covered by RUSP (19)
33	Disorders of nitrogen-containing compounds	HSD10 mitochondrial disease; HSD10MD	300438	A.14.15				Yes
34		Hyperornithinemia-hyperammonemia-homocitrullinuria syndrome; HHHS	238970	A.7.07			Yes	
35		Hyperphenylalaninemia, non-PKU mild, included	261600	A.11.01				Yes
36		Isovaleric acidemia; IVA	243500	A.14.07				Yes
37		Lesch-Nyhan syndrome; LNS	300322	A.2.11	Yes			
38		Lysinuric protein intolerance; LPI	222700	A.8.06			Yes	
39		Maple syrup urine disease; MSUD	248600	A.14.24				Yes
40		Methylmalonic aciduria due to methylmalonyl-CoA mutase deficiency	251000	A.14.21				Yes
41		Mitochondrial short-chain enoyl-CoA hydratase 1 deficiency; ECHS1D	616277	A.14.13			Yes	
42		Ornithine transcarbamylase deficiency, hyperammonemia due to	311250	A.7.03			Yes	
IEM #	Disease Types	IEM	OMIM	[a]IEMbase Nosology	Approach (No. of IEM)			
					Screened by Metabolomics (70 in total)			

© 2021 Liu N et al. *JAMA Network Open*.

IEM #	Disease Types	IEM	OMIM	[a]IEMbase Nosology	Approach (No. of IEM) Screened by Metabolomics (70 in total): Metabolomics Only (28 IEM)		Metabolomics and Traditional (42 IEM)	
					Metabolomics Only – NOT Covered by RUSP (26)	Metabolomics Only - Covered by RUSP (2)	Metabolomics & Traditional – NOT Covered by RUSP (23)	Metabolomics & Traditional - Covered by RUSP (19)
43	Disorders of nitrogen-containing compounds	Phenylketonuria; PKU	261600	A.11.01				Yes
44		Phosphoglycerate dehydrogenase deficiency; PHGDHD	601815	A.23.01			Yes	
45		Phosphoserine aminotransferase deficiency; PSATD	610992	A.23.02			Yes	
46		Propionic acidemia	606054	A.14.18, A.14.19				Yes
47		Spastic paraplegia 9B, autosomal recessive; SPG9B	616586	A.16.02			Yes	
48		Succinic semialdehyde dehydrogenase deficiency; SSADHD	271980	A.17.06			Yes	
49		Tyrosinemia, type I; TYRSN1	276700	A.12.07				Yes
50		Urocanase deficiency; UROCD	276880	A.18.02	Yes			
51	Disorders of peroxisomes and oxalate	Adrenoleukodystrophy; ALD	300100	H.110.01		Yes		
52		D-bifunctional protein deficiency	261515	H.110.03	Yes			
53		Peroxisome biogenesis disorder 1A (Zellweger); PBD1A	214100	H.112.01	Yes			

© 2021 Liu N et al. *JAMA Network Open*.

					Metabolomics Only (28 IEM)		Metabolomics and Traditional (42 IEM)	
					Metabolomics Only – NOT Covered by RUSP (26)	Metabolomics Only - Covered by RUSP (2)	Metabolomics & Traditional – NOT Covered by RUSP (23)	Metabolomics & Traditional - Covered by RUSP (19)
54	Disorders of peroxisomes and oxalate	Peroxisome biogenesis disorder 4A (Zellweger); PBD4A	614862	H.112.05	Yes			
55		Peroxisome biogenesis disorder 8A (Zellweger); PBD8A	614876	H.112.11	Yes			
56	Disorders of vitamins, cofactors and minerals	Brown-Vialetto-Van Laere syndrome 2; BVVLS2	614707	B.30.03			Yes	
57		Homocystinuria due to deficiency of n(5,10)-methylenetetrahydrofolate reductase activity	236250	B.27.03			Yes	
58		Homocystinuria-megaloblastic anemia, cblG complementation type; HMAG	250940	B.26.13			Yes	
59		Lipoyltransferase 1 deficiency; LIPT1D	616299	B.25.03			Yes	
60		Methylmalonic aciduria and homocystinuria, cblC type; MAHCC	277400	B.26.09				Yes
61		Multiple acyl-CoA dehydrogenase deficiency; MADD	231680	B.30.08				Yes
62		Multiple mitochondrial dysfunctions syndrome 1; MMDS1	605711	B.25.04	Yes			
IEM #	Disease Types	IEM	OMIM	[a]IEMbase Nosology	Approach (No. of IEM)			
					Screened by Metabolomics (70 in total)			
					Metabolomics Only		Metabolomics and Traditional	

© 2021 Liu N et al. *JAMA Network Open*.

					(28 IEM)		(42 IEM)	
					**Metabolomics Only – NOT Covered by RUSP (26)**	**Metabolomics Only - Covered by RUSP (2)**	**Metabolomics & Traditional – NOT Covered by RUSP (23)**	**Metabolomics & Traditional - Covered by RUSP (19)**
63	Disorders of vitamins, cofactors and minerals	Neurodegeneration, infantile-onset, biotin-responsive; NERIB	618973	B.26.17	Yes			
64	Mitochondrial disorders of energy metabolism	Developmental and epileptic encephalopathy 25, with amelogenesis imperfecta; DEE25	615905	D.55.09	Yes			
65		L-2-hydroxyglutaric aciduria; L2HGA	236792	D.55.02			Yes	
66		Liver failure, infantile, transient; LFIT	613070	D.70.12	Yes			
67		Mitochondrial complex V (ATP synthase) deficiency, nuclear type 2; MC5DN2	614052	D.67.01			Yes	
68		Mitochondrial DNA depletion syndrome 1 (MNGIE type); MTDPS1	603041	D.69.08	Yes			
69		Mitochondrial DNA depletion syndrome 9 (encephalomyopathic type with methylmalonic aciduria); MTDPS9	245400	D.55.05			Yes	
70		Pyruvate dehydrogenase, alpha-1; PDHA1	300502	D.54.01			Yes	

A complete list of 70 disorders screened by plasma clinical metabolomics is provided. Each disorder is categorized by one of 7 disease categories as defined by IEMBase v.2.0.0 and indicated as covered by either 1) the traditional screening approach (plasma amino acids, urine organic acids, and plasma acylcarnitine profile) and/or 2) the Recommended Uniform [Newborn] Screening Panel (RUSP). IEMbase v.2.0.0 was applied as the criterion for categorizing the conditions listed (http://www.iembase.org).

[a]IEMbase nosology number shown in this column for all conditions except isopentenyl-diphosphate delta isomerase 1; IDI1 for which a number is not available. Abbreviations: N/A: information is not available.

© 2021 Liu N et al. *JAMA Network Open*.
